# Synergistic Tuning of Conformational Dynamics, Electron Tunneling, and Substrate Positioning Enhances Electron Transfer in a P450 Chimera for Calcifediol Biosynthesis

**DOI:** 10.1002/advs.202519381

**Published:** 2026-01-20

**Authors:** Ziqi Liang, Xitong Song, Shuming Cheng, Yiwen Shen, Jie Zhang, Yaru Wang, Huiying Luo, Bin Yao, Binju Wang, Tao Tu

**Affiliations:** ^1^ State Key Laboratory of Animal Nutrition and Feeding Institute of Animal Sciences Chinese Academy of Agricultural Sciences Beijing P. R. China; ^2^ College of Animal Science and Technology Northwest A&F University Yangling Shaanxi P. R. China; ^3^ State Key Laboratory of Physical Chemistry of Solid Surfaces and Fujian Provincial Key Laboratory of Theoretical and Computational Chemistry College of Chemistry and Chemical Engineering Xiamen University Xiamen P. R. China; ^4^ Fujian Provincial Key Laboratory of Ecological Impacts and Treatment Technologies For Emerging Contaminants College of Environmental and Biological Engineering Putian University Putian P. R. China

**Keywords:** calcifediol biocatalysis, conformational, dynamics modulation, electron transfer, multi‐domain P450s engineering

## Abstract

Electron transfer (ET) efficiency dictates catalytic performance in multi‐domain self‐sufficient cytochrome P450s. Conventional engineering, however, predominantly focuses on localized optimization of either the active‐site pocket or linker regions, overlooking inter‐domain conformational transitions and ET chain integrity. Herein, we report a holistic ET‐optimization strategy integrating conformational dynamics modulation, ET pathway engineering, and substrate positioning tuning, which was applied to enhance ET in a chimeric P450 VK1‐CYP116B46‐L21 (L21) for calcifediol biosynthesis. [2Fe‐2S]→Heme ET pathway engineering yielded variant L21‐M2 (F346K/R354M), which decreased the conformational transition barrier by 4.5 kcal/mol and shortened the ET pathway by 4.05 Å, leading to a 72‐fold enhancement in ET rate. Heme domain engineering generated variant L21‐M3 (P83A/A177M/K180F), which shortened the near‐attack conformations distance to 4.19 Å (from 4.62 Å) and increased reactive conformation to 38 % (from 25 %). The pentuple variant L21‐M5 combined both improvements, which demonstrated exceptional catalytic performance: an 8.2‐ fold higher catalytic efficiency, a coupling efficiency of 56.78 %, and a total turnover number (TTN) of 3222. In a semi‐preparative‐scale biotransformation, L21‐M5 achieved 3.26 g/L production of calcifediol with 82 % conversion, underscoring its strong industrial potential. These results highlight the efficacy of the proposed ET‐optimization strategy and provide a transferable workflow for engineering multi‐domain redox biocatalysts.

## Graphical abstract

A synergistic electron transfer (ET) optimization strategy modulating conformational dynamics, ET pathway, and substrate orientation is proposed for self‐sufficient cytochrome P450s. Guided by this strategy, a P450 variant with improved ET and catalytic efficiency was identified, and the underlying molecular basis was revealed by computational analysis. This work provides a transferable workflow for engineering multi‐domain redox biocatalysts.

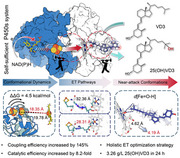

## 1 Introduction

Cytochrome P450 monooxygenases (P450s) stand out as the most versatile biocatalysts in nature, mediating regio‐ and stereoselective oxidative reactions under mild physiological conditions [1, 2]. This unique capability has positioned them as pivotal tools in drug synthesis and natural product diversification [3]. However, the P450s catalytic cycle relies strictly on a sequential electron transfer (ET) process, introducing critical bottlenecks: low coupling efficiency (where electrons are wasted on unproductive side reactions), accumulation of reactive oxygen species (ROS) that inactivate the enzyme, and sluggish turnover rates [4, 5, 6, 7]. Addressing these limitations—by understanding and enhancing ET efficiency—has thus emerged as a central challenge in P450s engineering.

The P450s catalytic cycle initiates with a low‐spin heme‐H_2_O complex [6]. Substrate binding displaces the axial water molecule, triggering a shift in the heme iron to a high‐spin Fe^III^ state. This spin transition elevates the reduction potential of iron, generating a thermodynamic driving force for ET from redox partners [8]. Subsequent steps include the reduction of Fe^III^ to Fe^II^, binding of O_2_ to form a Fe^II^‐O_2_ adduct, and a subsequent ET coupled with protonation to generate the highly reactive Fe═O porphyrin cation radical (Compound I, Cpd I)—the species directly responsible for substrate oxidation [9]. For non‐native substrates, two key defects often compromise efficiency: (i) improper substrate positioning, which increases the distance between the Cpd I Fe═O moiety and the substrate's reactive hydrogen (*d*[Fe = O─H])—disrupting the. radical rebound mechanism and promoting uncoupling [10]; and (ii) inadequate substrate‐induced spin‐state switching, which weakens the “reduction‐potential switch” and leads to electron leakage [11, 12, 13]. These observations highlight that ET efficiency in P450s is governed by two interconnected factors: (i) the efficiency of electron delivery from reductase domains to the heme center, and (ii) the capacity of the heme domain to utilize delivered electrons for productive catalysis.

**FIGURE 1:**
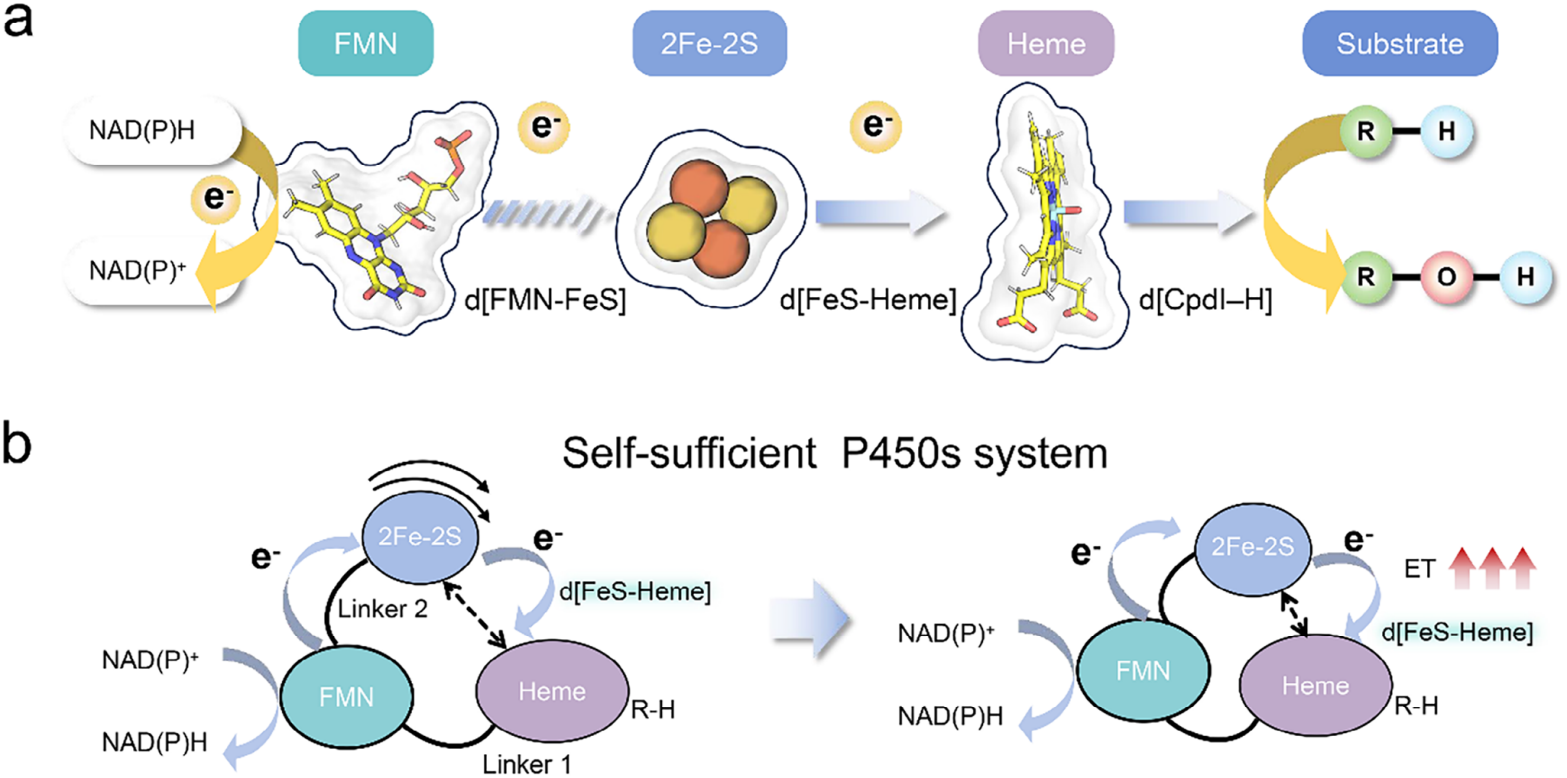
(a) Schematic of the typical ET pathway in a self‐sufficient P450 system during hydroxylation reactions. FMN: flavin mononucleotide; [2Fe‐2S]: iron sulfur cluster. The distances between FMN and the [2Fe‐2S] cluster, between the [2Fe‐2S] cluster and heme, and between the Cpd I Fe═O atom and the substrate reactive hydrogen are denoted as *d*[FMN‐FeS], *d*[FeS‐Heme], and *d*[Fe═O─H], respectively. (b) Substrate binding triggers a reversible distal‐to‐proximal conformational change in the ferredoxin domain of CYP116B46, resulting in a shortened *d*[FeS‐Heme] and enhanced ET efficiency.

Most P450s rely on one or two soluble redox partners (e.g., ferredoxins, flavoproteins) to complete their catalytic cycle [14], but the requirement for exogenous partners complicates their application in biocatalysis. To address this limitation, recent engineering efforts have focused on self‐sufficient P450s—enzymes that fuse the monooxygenase (heme) domain and redox (flavin/iron‐sulfur) domains into a single polypeptide [4, 9, 15, 16]. Examples such as P450BM3 (FAD/FMN‐dependent) and CYP116B46 ([2Fe‐2S]/FMN‐dependent) leverage intramolecular ET to shorten electron tunneling distances, reduce energy barriers, and minimize uncoupling [17, 18]. A defining feature of these multi‐domain systems is that ET efficiency is tightly regulated by the conformational dynamics of redox domains (Figure 1a,b). In P450BM3, for instance, the FMN‐binding domain undergoes a ∼10 Å hinge‐mediated shift upon substrate binding, reducing the distance to the heme iron from 18.4 Å (substrate‐free state) to a catalytically competent range [11]. Similarly, in CYP116B46, umbrella sampling molecular dynamics (MD) simulations and ET pathway calculations have shown that the [2Fe‐2S]‐containing ferredoxin domain undergoes a distal‐to‐proximal rotational transition. This transition shortens the [2Fe‐2S]‐to‐heme distance (*d*[FeS–Heme]) and enhances ET along the NAD(P)H→heme chain (Figure 1b) [19]. Collectively, these studies demonstrate that conformational transitions are a universal rate‐limiting step in self‐sufficient P450s; when substrate‐induced or linker‐mediated domain motions are inadequate, ET efficiency declines drastically [20, 21].

Conventional protein engineering has predominantly focused on scanning “hotspots” within catalytic pockets [22, 23, 24] and substrate access channels [25], where beneficial mutations are identified and combined to refine local catalytic properties. This “rigid scaffolding” paradigm, however, overlooks the conformational dynamics of multi‐domain P450s—their catalytic performance arises from intricate networks of transient interdomain interactions, long‐range conformational rearrangements, and context‐dependent cofactor dynamics [26, 27]. Incorporating this dynamic perspective is therefore indispensable in protein engineering—particularly for large, multi‐domain enzymes, whose function hinges on coordinated motions between spatially distant structural elements [28]. Advancing P450s engineering thus requires moving beyond localized active‐site hotspot screening to elucidate global conformational dynamics and interdomain interaction networks.

25‐hydroxyvitamin D3 (25(OH)VD3, calcifediol) is a clinically critical metabolite used to treat rickets, osteoporosis, and vitamin D deficiency [29, 30, 31]. Self‐sufficient P450s have emerged as promising biocatalysts for 25(OH)VD3 biosynthesis via regioselective C25 hydroxylation of VD3 [23, 32], but their performance remains limited by the aforementioned ET bottlenecks. Previous attempts to optimize chimeric self‐sufficient P450s have focused on linker engineering—truncating or mutating the flexible regions connecting heme and reductase domains to modulate domain dynamics [33, 34]. In our prior work [35], we constructed chimeric self‐sufficient P450s by fusing the heme domain of Vdh‐K1 (a VD3‐hydroxylating P450) with the redox domain of CYP116B46. From these constructs, we identified two constructs—VK1‐CYP116B46‐L13 (L13) and VK1‐CYP116B46‐L21 (L21)—that differ by only 8 linker residues yet exhibit a 10‐ fold activity difference in activity (L21> L13). The structure–function relationship between this activity discrepancy and conformational dynamics, however, remains to be elucidated.

In this study, we used L21 as a model to improve its ET efficiency through a comprehensive strategy integrating conformational dynamics, ET pathways, and substrate specificity. We first performed a semi‐rational design of the [2Fe‐2S]→heme pathway in L21, guided by insights into conformational dynamics and the ET pathway. This effort identified the dual variant F346K/R354M (designated L21‐M2), which exhibits improved coupling efficiency and activity. Computational analysis revealed that, compared with L13 and L21, L21‐M2 has a reduced energy barrier for the distal‐to‐proximal conformational transition of the redox domain and a 4.05 Å shortening of the ET pathway. We then conducted a semi‐rational design on the heme domain's substrate pocket to improve substrate specificity and electron acceptance, resulting in the triple variant P83A/A177M/K180F (designated L21‐M3), which displays a 3‐ fold higher activity than L21. Computational analysis confirmed that L21‐M3 has a shortened *d*[Fe═O─H]. Combining the mutations from L21‐M2 and L21‐M3 yielded the pentuple variant L21‐M5, which exhibited further enhancements in ET efficiency and turnover—validating the effectiveness of our engineering strategy. Finally, we demonstrated the industrial applicability of L21‐M5 in 25(OH)VD3 biosynthesis via semi‐preparative‐scale biotransformation. Overall, this study presents generalizable strategies to boost ET efficiency in self‐sufficient P450s and clarifies the underlying molecular principles, paving the way for the rational design of multi‐domain enzymes with superior ET efficiency and their practical application in biotransformation processes.

## 2 Results and Discussion

### 2.1 Linker Flexibility, Not Static *d*[FeS‐heme], Governs ET Efficiency in Self‐Sufficient P450 Chimeras

**FIGURE 2:**
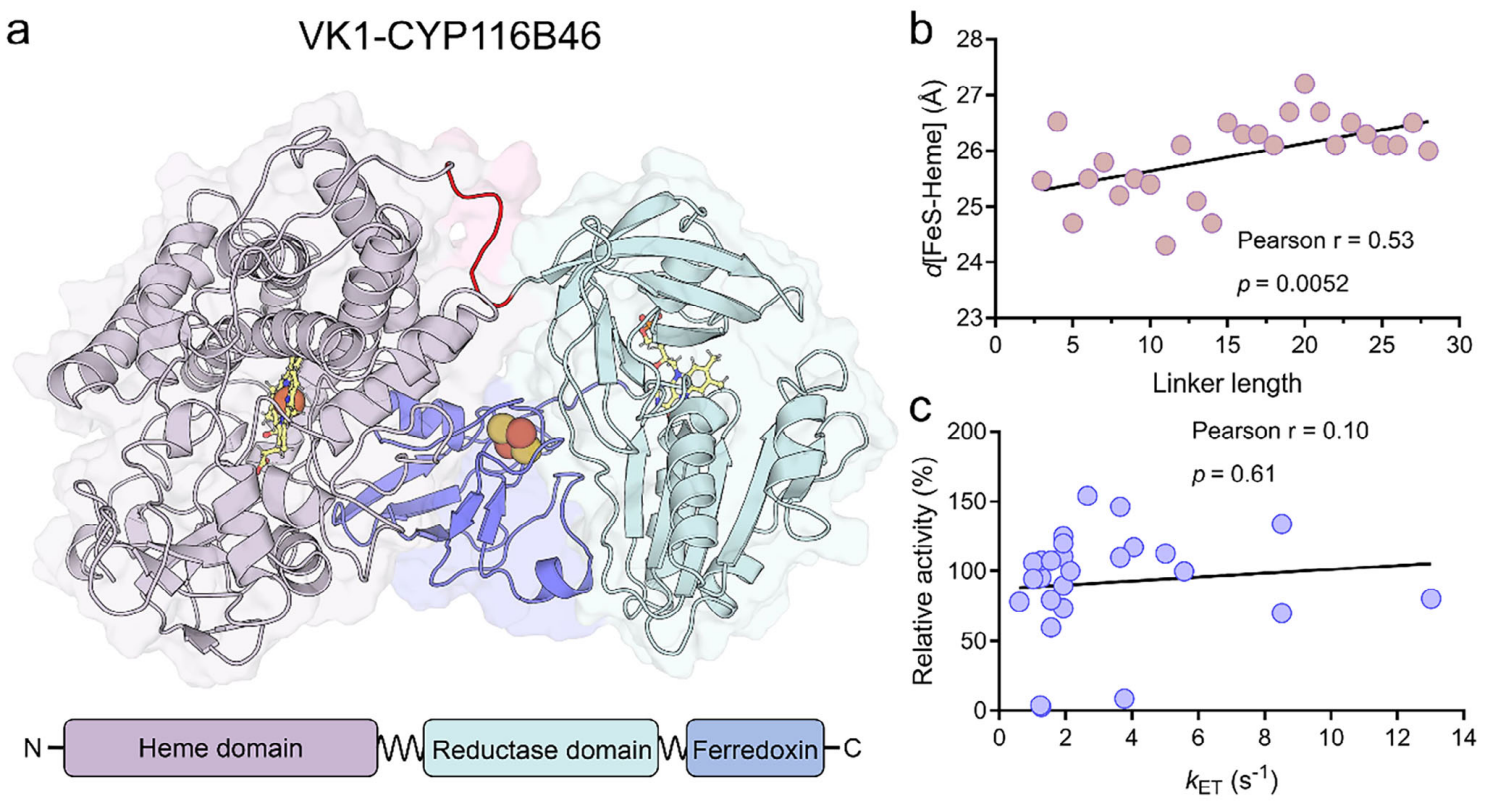
(a) Architecture of the engineered fusion protein VK1‐CYP116B46‐L21 (L21), constructed by fusing the heme domain from Vdh‐K1 (light red) with the reductase domain (cyan) and ferredoxin domain (blue) of CYP116B46, connected via a truncated native linker (red). (b) Pearson correlation analysis between linker length and *d*[FeS‐Heme] in 26 self‐sufficient P450 chimeras. (c) Pearson correlation analysis between ET rate (*k*_ET_) and relative activity. Results shown are representative of three biological replicates. Significance levels were defined as *p*< 0.05.

According to Marcus' theory [36], long‐range biological ET is strongly dependent on the electronic coupling matrix element (VDA) between the electron donor and acceptor—a parameter highly sensitive to the spatial separation of these two moieties [37, 38, 39]. To investigate the structure‐function relationship between ET distance and activity of self‐sufficient P450s, we used AlphaFold3 [40] to model the structures of 26 previously constructed chimeric P450s. These chimeras were generated by fusing the heme domain of Vdh‐K1 with the redox domain of CYP116B46, using truncated natural linkers of varying lengths (Figure 2a) [35]. In the crystal structure of CYP116B46 (PDB ID: 6LAA), the *d*[FMN‐FeS] is 7.9 Å—a separation that enables efficient ET from FMN to [2Fe‐2S] cluster [19], and we therefore analyzed the relatively longer *d*[FeS‐heme].

Our modeling results showed that *d*[FeS‐heme] across the 26 chimeras ranged from 24.3 to 27.2 Å (Figure 2b), and a stable and moderately strong positive correlation (Pearson *r* = 0.53, *p* = 0.0052) with linker length, meaning that longer linkers tend to correspond to greater distances. However, only a weak and non‐significant negative trend was observed between catalytic activity and the *d*[FeS‐heme] distance (*r* = −0.20, *p* = 0.32) (Figure S1). We further calculated the ET rate (*k*_ET_) with Equation 5 and found that no significant linear association was found between *k*_ET_ and catalytic activity (*r* = 0.10, *p* = 0.61) (Figure 2c). This result demonstrates that static interdomain distance (*d*[FeS‐heme]) alone is insufficient to explain the observed differences in catalytic activity among the chimeras. Collectively, these results demonstrate that when variations in static interdomain distance are minimal, ET efficiency in self‐sufficient P450 chimeras is governed by the protein's global transient conformational dynamics. This insight further suggests that “dynamic hotspots” should not be restricted to linker regions; domain–domain interfaces also represent key targets for modulating conformational flexibility—and thus ET efficiency—in multi‐domain P450s.

### 2.2 Enhancing ET Rates Through Semi‐Rational Design of the [2Fe‐2S]→Heme Pathway in L21

**FIGURE 3:**
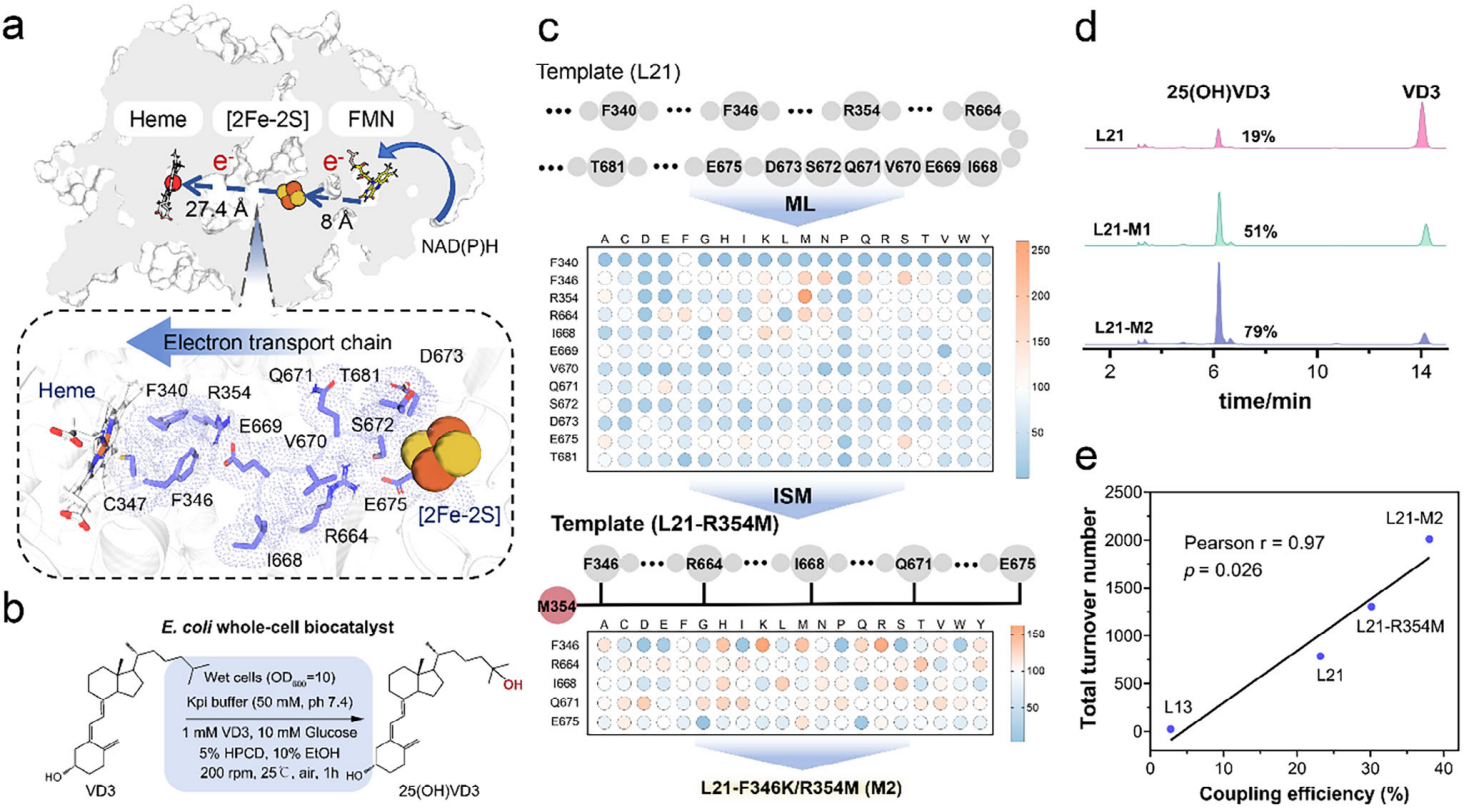
Engineering the ET pathway from the [2Fe‐2S] cluster to heme in L21. (a) In L21, the distances *d*[FMN‐FeS] and *d*[FeS‐Heme] are 8.0 and 27.4 Å, respectively. 12 residues potentially involved in mediating ET between the [2Fe‐2S] cluster and heme are highlighted. (b) Schematic of the whole‐cell biocatalysis system for C25‐hydroxylation of VD3 using *E. coli* cells. Reaction conditions: recombinant *E. coli* cells frozen at ‒80°C were resuspended in potassium phosphate buffer (50 mM, pH 7.4, OD_600_ = 10) containing 1 mM VD3, 10 mM glucose, 5 % (w/v) hydroxypropyl‐β‐cyclodextrin (HPCD), and 10 % (v/v) ethanol as a cosolvent. Reactions were carried out at 25°C and 200 rpm for 1 h. (c) Protein engineering strategy applied to L21. MLs were constructed for residues F340, F346, R354, R664, E669, V670, Q671, S672, D673, E675, and T681. Variant L21‐R354M was selected as the template for ISM at positions F346, R664, I668, Q671, and E675. (d) Representative HPLC chromatograms showing VD3 conversion catalyzed by L21 and selected variants. (e) Pearson correlation analysis between TTN and coupling efficiency for L13, L21, and their variants. Results shown are representative of three biological replicates. Significance levels were defined as *p* < 0.05.

To explore the structure–function relationship between inter‐domain conformational flexibility and ET rate, we chose the most active chimeric L21 as the model system. The structure of L21 was superimposed onto the crystal structure of CYP116B46 with a root‐mean‐square deviation (RMSD) of 0.121 Å, indicating high structural similarity between the enzymes (Figure S2a). In L21, the distance between FMN and the [2Fe‐2S] cluster (*d*[FMN‐FeS] = 8.0 Å) is considerably shorter than that between the [2Fe‐2S] cluster and the heme group (*d*[FeS‐Heme] = 27.4 Å) (Figure 3a). To increase the ET rates of the [2Fe‐2S]→Heme pathway in L21, we conducted protein engineering from two aspects: conformational dynamics and the ET pathway. Previous analysis of CYP116B46 using the “Pathways” plugin identified Q384, the E723–S726 loop, and D727 as key residues in the ET pathway (Figure S2b) [19, 41]. In L21, the equivalent positions (F346, E669–S672), together with residues near the cofactors (F340, D673, E675, and T681) and three domain–domain interface residues (R354, R664, and I668), totaling 12 sites, were selected for mutagenesis library analysis (MLs; Figure 3a).

Variants were co‐expressed with glucose dehydrogenase (GDH; PDB ID: 3AUS) in *E. coli* BL21 to enable NAD(P)H regeneration for whole‐cell biocatalysis screening (Figure 3b; Figure S3). The results revealed that substitutions at F340, E669, V670, S672, D673, and T681 generally decreased enzymatic activity (Figure 3c). Notably, mutations at F340 nearly abolished activity, likely due to its critical role in heme binding—a corresponding F378A mutation in CYP116B46 was previously shown to eliminate CO‐binding capacity [41]. In contrast, beneficial mutations were identified at F346, R354, R664, I668, Q671, and E675. Specifically, F346M, R354M, and R664M increased activity by 62 %, 160 %, and 53 %, respectively, highlighting the potential role of methionine in facilitating multi‐step ET [42]. The variant L21‐R354M, for instance, increased conversion from 19 % to 51 % compared to L21. Subsequently, iterative saturation mutagenesis (ISM) was applied to sites F346, R664, I668, Q671, and E675 on the L21‐M1 background. HPLC screening identified F346K/R354M and F346R/R354M as the top‐performing variants, exhibiting 62 % and 55 % higher activity than L21‐R354M, respectively. The variant F346K/R354M (designated L21‐M2) achieved 79 % conversion (Figure 3d).

To further characterize the engineered variants, L21 and its variants were purified for kinetic analysis (Table 1; Figure S8). L21‐M2 exhibited the highest catalytic efficiency (*k*_cat/_ *K*_m_; 36.14 mM^−1^ s^−1^), representing a 3.4‐ fold improvement over L21, along with a 156 % increase in TTN. The coupling efficiency also increased from 23.19 % in L21 to 38.03 % in L21‐M2. A significantly strong positive correlation (Pearson *r* = 0.97, *p* = 0.026, Figure 3e) was observed between TTN and coupling efficiency, confirming that enhanced ET efficiency directly contributes to improved TTN. These results demonstrate that rational redesign of ET pathway residues can effectively modulate ET efficiency.

**TABLE 1:** Kinetic parameters, TTN, and coupling efficiency of L13, L21, and L21 variants.

Enzyme	*K*_m_ (mM)	*k*_cat_ (s^−1^)	*k*_cat_/*K*_m_ (mM^−1^ s^−1^)^a^	TTN^b^	Coupling efficiency (%)^c^
L13 [35]	0.17 ± 0.04	0.14 ± 0.01	0.79	25	2.78
L21	0.18 ± 0.04	1.47 ± 0.08	8.23	786	23.19
L21‐R354M	0.18 ± 0.03	2.99 ± 0.15	16.85	1306	30.13
L21‐F346K/R354M (L21‐M2)	0.16 ± 0.03	5.71 ± 0.22	36.14	2011	38.03
L21‐P83A/A177M/K180F (L21‐M3)	0.14 ± 0.02	4.81 ± 0.20	34.50	1573	36.34
L21‐P83A/A177M/K180F/F346K/R354M (L21‐M5)	0.12 ± 0.02	9.21 ± 0.29	75.92	3222	56.78

^a^ Reaction conditions: purified enzymes (1 µM) in potassium phosphate buffer (50 mM, pH 7.4), NADH (5 mM), VD3 (0.01–5 mM), 25°C, 1000 rpm for 10 min. Results shown are representative of three biological replicates.

^b^ TTN: purified enzymes (0.2 µM) in potassium phosphate buffer (50 mM, pH 7.4), NADH (5 mM), VD3 (1 mM), 25°C, 200 rpm for 4 h.

^c^ Coupling efficiency: purified enzymes (1 µM), 1 mM VD3, and 1 mM NADH, 25°C, 1000 rpm until NADH was fully consumed.

### 2.3 Elucidating the ET Mechanism in L21‐M2

**FIGURE 4:**
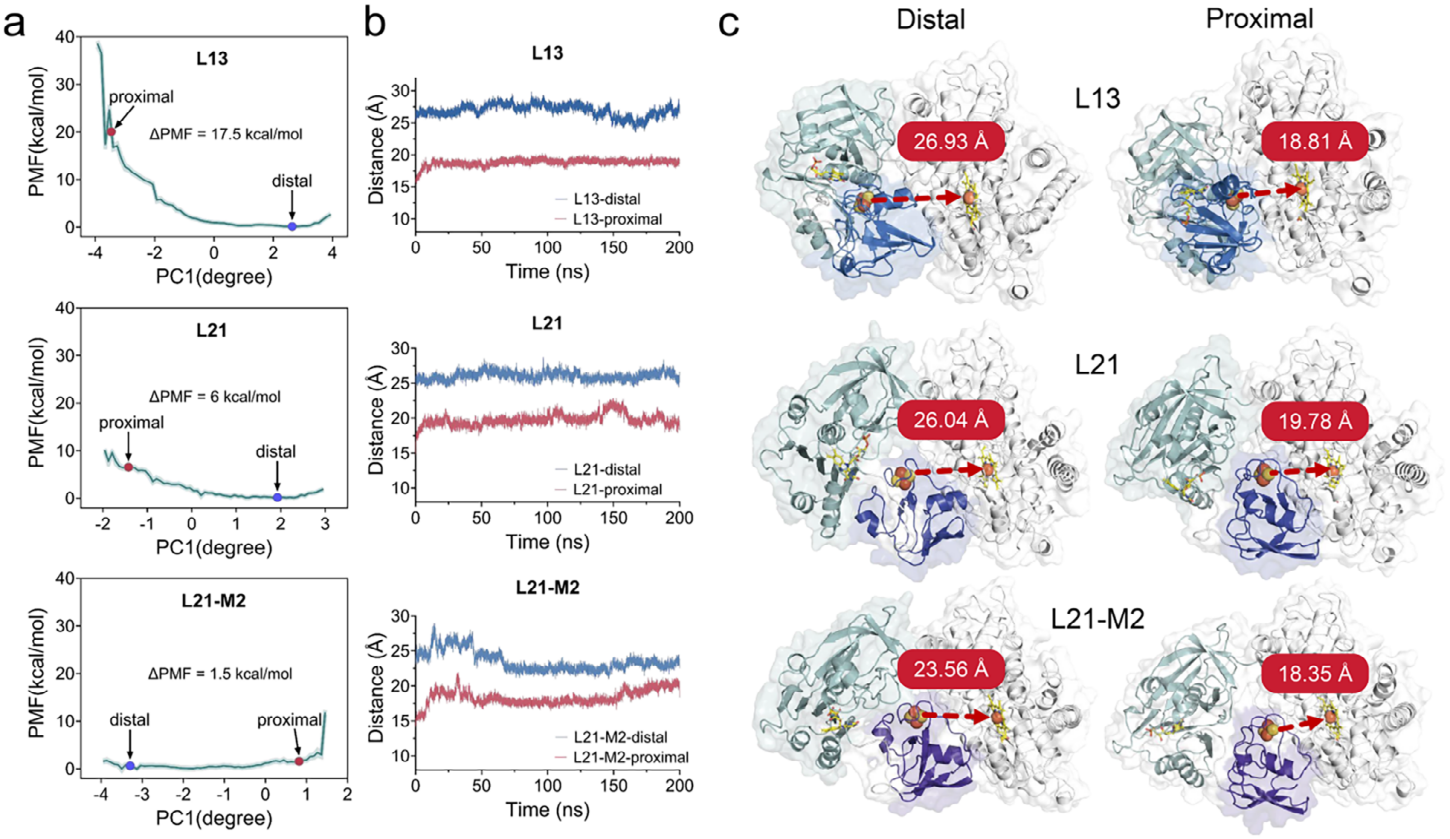
Conformational energy landscape and intercofactor distance dynamics derived from three replicates of umbrella sampling simulations. (a) PCA‐based analysis of the PMF for projected conformational transitions. The positions of the distal and proximal states are indicated by black arrows. (b,c) Time‐dependent changes of *d*[FeS‐Heme] during 200 ns unrestricted MD simulations for the distal and proximal conformations of L13, L21, and L21‐M2.

In self‐sufficient cytochrome P450 systems, the dynamics of redox domains are often rate‐limiting. Comparative analysis of conformational dynamics between wild type and variants is essential for understanding enhancements in catalytic activity and coupling efficiency. Although L13 and L21 differ by only 8 linker residues, L13 exhibits markedly reduced activity, underscoring the critical influence of linker flexibility on redox domain dynamics. Attempts to determine the structures of L13, L21, and L21‐M2 using cryo‐electron microscopy were hindered by their relatively small size (81 kDa) and monomeric state in solution. We therefore employed MD simulations. Structural models of L13 and L21‐M2 were generated using AlphaFold3, with cofactors (FMN, [2Fe−2S]) and substrate VD3 docked via Smina [43]. During 200 ns MD simulations of distal conformations, the average *d*[FeS‐Heme]) was slightly shorter in L21‐M2 (23.56 Å) than in L21 (26.04 Å) or L13 (26.93 Å); however, this minor difference alone could not account for the substantial divergence in catalytic activity. To probe the thermodynamic relationship between catalytic performance and conformational transitions, we employed umbrella sampling combined with principal component analysis (PCA) to reconstruct the free energy landscapes associated with these structural changes (Figure 4a; Figures S5–S7). The energy barrier for transitioning from the distal to proximal conformation was significantly lower in L21‐M2 (∼1.5 kcal/mol) compared to L21 (∼6 kcal/mol) and L13 (∼17.5 kcal/mol) (Figure 4a), indicating a reduction in the thermodynamic obstacle to conformational rearrangement. Subsequent 200 ns unrestricted MD simulations of the proximal conformations yielded stable structural states (Figure 4b,c). In these proximal conformations, the average *d*[FeS‐Heme]) were 18.81, 19.78, and 18.35 Å for L13, L21, and L21‐M2, respectively. Collectively, these results suggest that the low catalytic activity of L13 originates from a considerably higher free energy barrier for interconversion between distal and proximal conformations.

**FIGURE 5:**
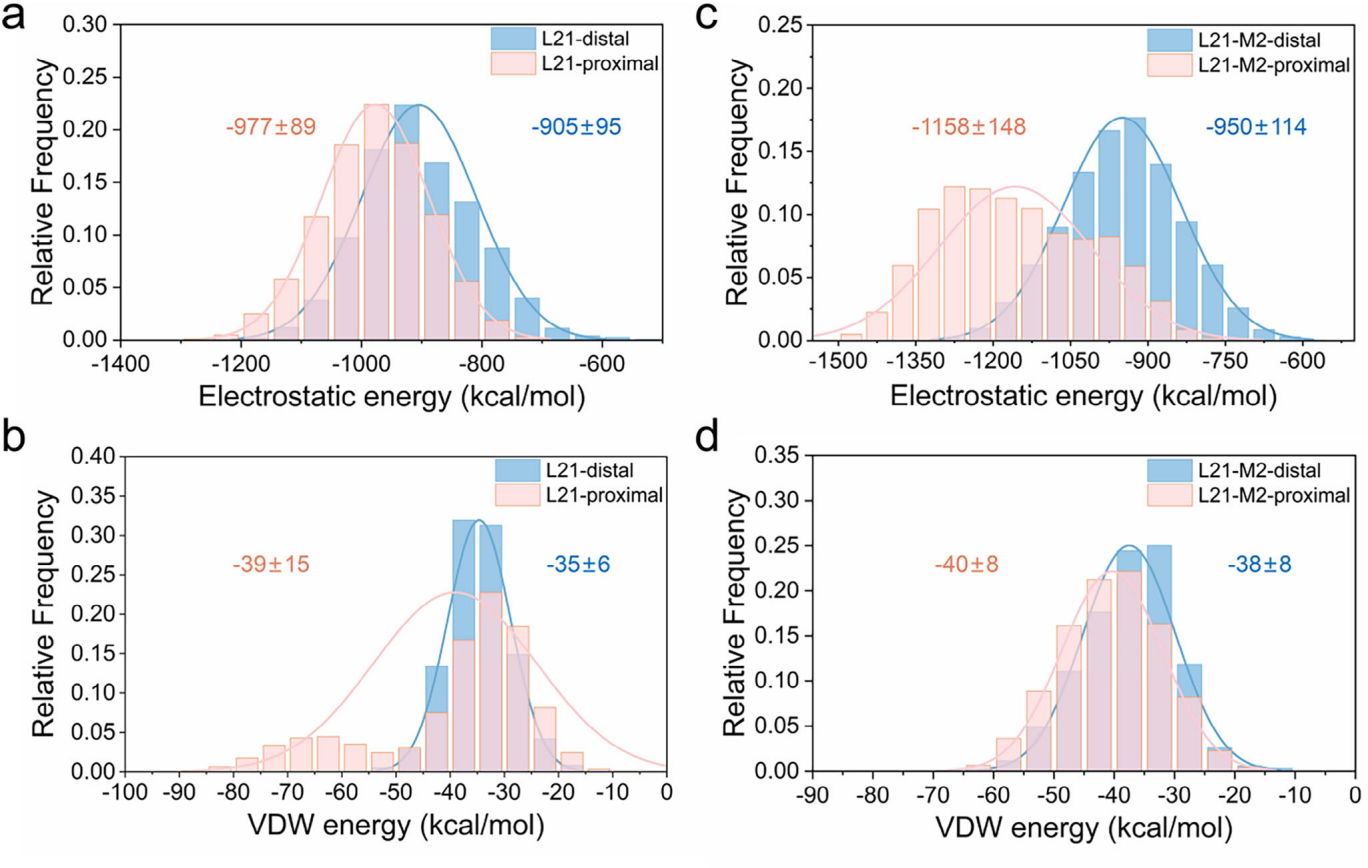
Statistics of nonbonded interactions between the ferredoxin domain and the other two domains in L12 and L12‐M2. Energy distributions are shown for distal (blue) and proximal (red) conformations, computed using a cutoff distance of 99 Å to include long‐range electrostatic effects. (a) Electrostatic and (b) vdW interaction energies for L21. (c) Electrostatic and (d) vdW interaction energies for L21‐M2. Mean values ± standard deviation are indicated above each distribution.

To investigate whether the facilitated conformational transition in L21‐M2 correlates with changes in nonbonded interaction energies, we analyzed the electrostatic and van der Waals (vdW) energy distributions between the ferredoxin domain and the other two domains across different conformational states. A generous cutoff distance of 99 Å was applied to ensure the inclusion of long‐range interactions, particularly electrostatic effects [19]. Relative to the distal conformations, the proximal conformations in L21 and L21‐M2 exhibited strengthened electrostatic interactions and vdW interactions (Figure 5), suggesting that the transition to the proximal conformation is primarily driven by nonbonded interactions—especially electrostatic forces. Furthermore, no significant difference in vdW interaction energies was observed in the proximal conformations between L21 and L21‐M2 (ranging from ‒39 ± 15 to ‒40 ± 8 kcal/mol) (Figure 5b,d). Notably, the electrostatic energy distribution in the proximal conformation of L21‐M2 shifted toward more negative values compared to L21 (from ‒977 ± 89 to ‒1158 ± 148 kcal/mol, Figure 5a,c), indicating enhanced electrostatic complementarity between the ferredoxin domain and the other domains. These results suggest that the stronger electrostatic interaction in the proximal conformation of variant L21‐M2 reduces the free energy barrier for conformational transition.

**FIGURE 6:**
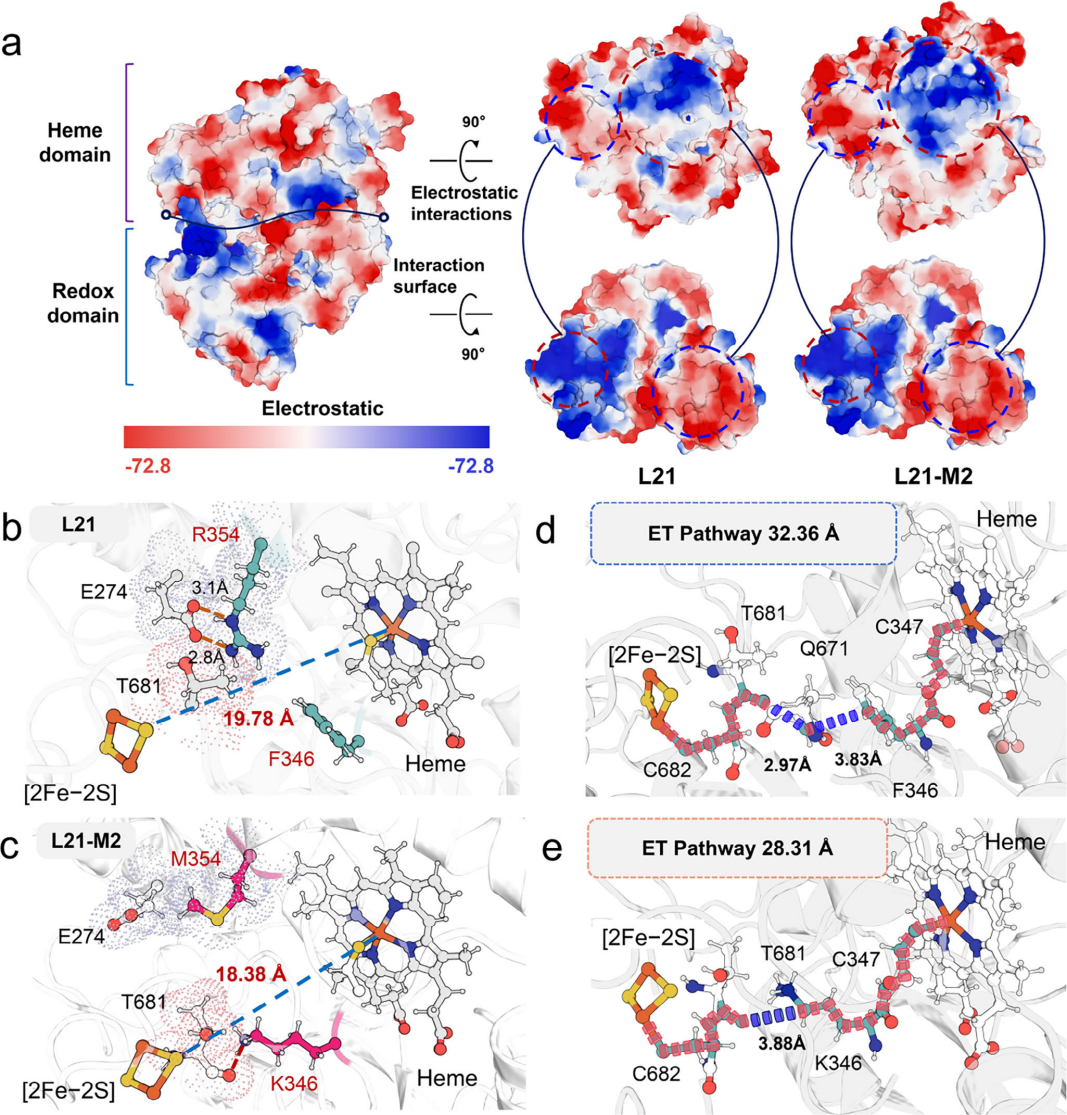
Structural and ET pathway analysis of the heme and ferredoxin domains in the proximal conformations of L21 and L21‐M2, respectively. (a) Electrostatic surface potential analysis of the heme domain and redox partner domain. The color scale represents electrostatic potential (red: negative; blue: positive). (b,c) Close‐up views of the interdomain interface in L21 b) and L21‐M2 (c), highlighting key interactions: salt bridges (orange dashed lines), hydrogen bonds (red dashed lines), and the distance between the [2Fe–2S] cluster and heme iron (*d*[FeS–Heme], blue dashed line). (d,e) Predominant ET pathways from the [2Fe–2S] cluster to the heme cofactor in L21 d) and L21‐M2 (e), where covalent tunneling steps are indicated by red dashed lines and through‐space tunneling by blue dashed lines.

Electrostatic surface potential analysis of representative MD snapshots revealed that the heme domain of L21‐M2 carries a markedly more positive surface at the ferredoxin interface compared to L21, strengthening interdomain attraction (Figure 6a). Additional structural inspection confirmed that, in L21, R354M in the heme domain forms a stable salt bridge with the adjacent E274, leading to steric hindrance between R354 and T681 in the ferredoxin domain, which impedes the close approach of the [2Fe‐2S] cluster to the heme (Figure 6b). The R354M mutation disrupts this salt bridge, allowing conformational rearrangement of the side chain and reducing steric conflict with T681 (Figure 6c). Simultaneously, the F346K mutation introduces a hydrogen bond (occupancy: 37.28 %) between the lysine side‐chain nitrogen and the main‐chain oxygen of T681, further stabilizing a catalytically productive inter‐domain orientation. Together, these mutations shorten the *d*[FeS‐Heme] from 19.78 Å in L21 to 18.38 Å in L21‐M2. Further structural analysis indicated that, in L21, residue R354 in the heme domain forms a relatively strong salt bridge with the neighboring E274. This interaction induces a steric clash between R354 and T681 in the ferredoxin domain, hindering the close approach of the [2Fe‐2S] cluster to the heme center. The R354M mutation disrupts this salt bridge, enabling side‐chain rearrangement of the mutated residue and alleviating the steric hindrance with T681. In parallel, the F346K mutation introduces a hydrogen bond between the lysine side‐chain nitrogen and the main‐chain oxygen of T681 (occupancy: 37.28 %), further stabilizing a productive inter‐domain orientation. Collectively, these mutations reduce the *d*[FeS‐Heme] from 19.78 Å in L21 to 18.38 Å in L21‐M2.

Using the “Pathways” plugin [44], we identified the most favorable ET routes from the [2Fe‐2S] cluster to the heme iron from representative MD snapshots of L21 and L21‐M2 (Figure 6d,e). In L21, ET proceeds via covalent tunneling from the [2Fe‐2S] cluster to T681, a through‐space jump to Q671, subsequent transfer to F346, and finally via covalent bonds through C347 to the heme iron, covering a total distance of 32.36 Å. In L21‐M2, after reaching T681, ET occurs via a through‐space tunneling event directly to K346, followed by transfer to the heme iron. This modified pathway results in a shorter total distance of 28.31 Å—a reduction of 4.05 Å that enhances electronic coupling (Figure 6e). Calculations of the ET rate (*k*_ET_) yielded values of 2.54 × 10^−3^ s^−1^ for L21 and 1.86 × 10^−1^ s^−1^ for L21‐M2, corresponding to a 72‐ fold increase in ET efficiency for the variant. These results demonstrate that engineering the ET pathway between the [2Fe‐2S] cluster and heme in L21‐M2 enhances ET efficiency by two key mechanisms: (i) reducing the energy barrier for conformational reorganization through strengthened electrostatic interactions, and (ii) shortening the electron tunneling distance while decreasing the number of hopping steps. Together, these optimizations improve both ET efficiency and coupling efficiency.

### 2.4 Improving Electron Acceptor Efficiency Through Evolution of the L21 Heme Domain

**FIGURE 7:**
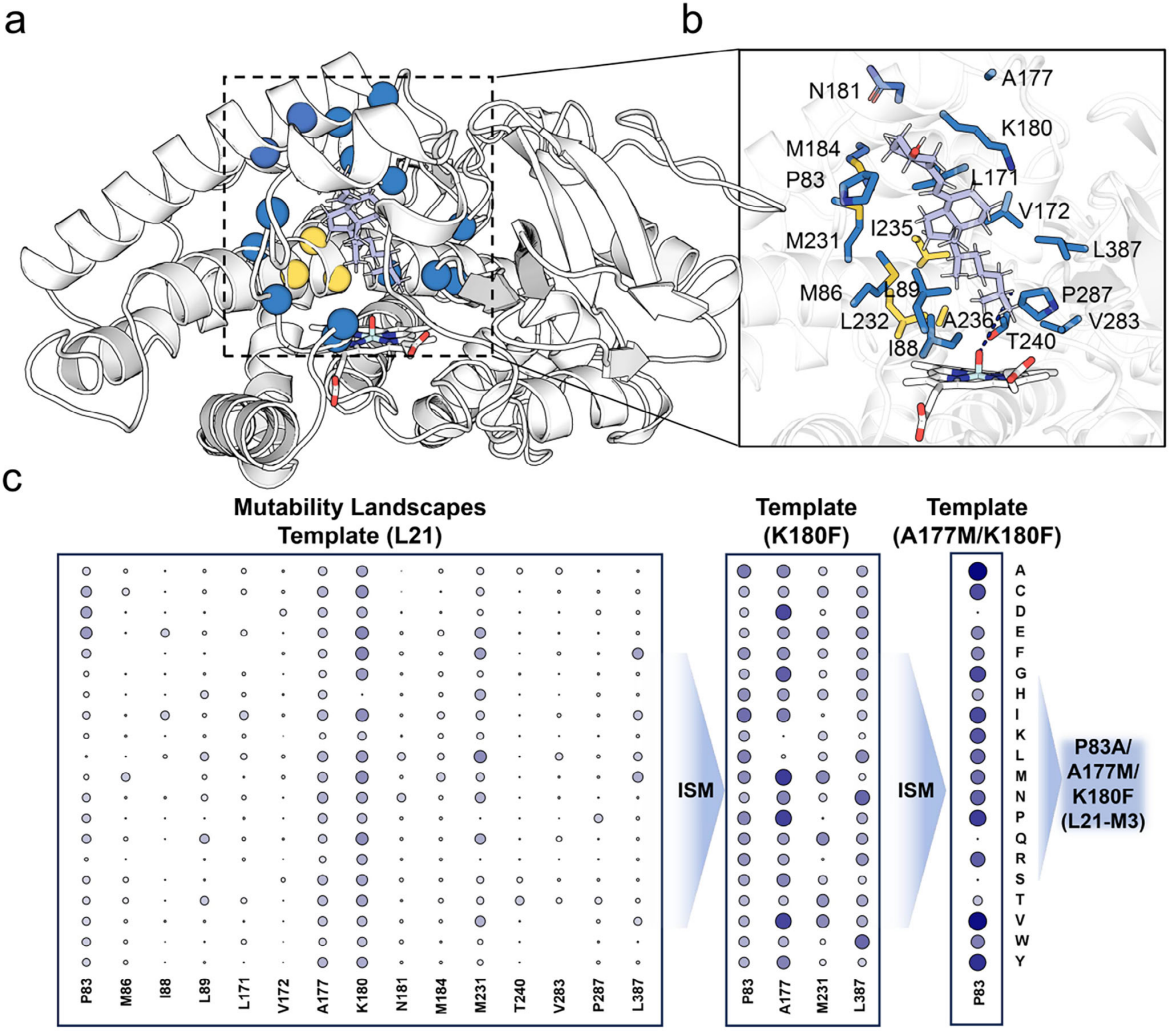
Improving electron acceptance through evolution of the L21 heme domain. (a) Representative MD stimulation snapshots of the Vdh‐K1‐VD3 complex (PDB ID:3A50) after 200 ns. (b) Key residues located in the VD3 binding pocket. The dashed line represents the *d*[Fe═O─H]. (c) Protein engineering strategy applied to the L21 heme domain. MLs were constructed at residues P83, M86, I88, L89, L171, V172, A177, K180, N181, M184, M231, T240, V283, P287, and L387. The variant K180F was identified as beneficial and used as the template for the first round targeting P83, A177, M231, and L387. The double variant A177M/K180F, selected from the first ISM round, served as the template for the subsequent round, which yielded the best triple variant P83A/A177M/K180F (L21‐M3). Results shown are representative of three biological replicates.

The heme group serves as the terminal electron acceptor in cytochrome P450 enzymes [16]. Proper substrate positioning within the active site is essential to facilitate an effective reduction‐potential switch and maintain an optimal reactive distance for *d*[Fe═O─H], thereby minimizing the waste of reducing equivalents and suppressing uncoupling reactions [45, 46]. To further improve electron acceptance and increase coupling efficiency, we conducted protein engineering on the heme domain of L21, aiming to shorten *d*[Fe═O─H] and enhance electron utilization. We initiated our study with a 200 ns MD simulation using the crystal structure of the Vdh‐K1‐VD3 complex (PDB ID: 3A50) [47] as the starting model. Snapshots exhibiting converged RMSD values were selected for subsequent analysis (Figure 7a). As shown in Figure 7b, VD3 resides in a hydrophobic pocket above the heme and is primarily stabilized by hydrophobic interactions. The initial distance *d*[Fe═O─H] was measured to be 5.2 Å (Figure 7b). Given that the oxygen‐binding motif residues L232, I235, and A236 are evolutionary conserved and critical for oxygen binding to the heme domain [48], we targeted 15 surrounding substrate‐interacting residues (P83, M86, I88, L89, L171, V172, A177, K180, N181, M184, M231, T240, V283, P287, and L387) for MLs construction, resulting in a total of 285 variants. Among these, only substitutions at P83, A177, K180, M231, and L387 led to increased activity (Figure 7c). With the exception of L387, no beneficial mutations were identified near the heme, underscoring the importance of hydrophobic interactions for VD3 binding.

Notably, all substitutions at K180 except K180H resulted in elevated activity, suggesting that the long polar side chains of lysine and histidine hinder the deep penetration of VD3 into the binding pocket. Among these variants, K180F exhibited the highest activity improvement (126 %) and was selected as the template for ISM at P83, A177, M231, and L387, yielding a focused library of 76 variants. Screening revealed beneficial mutations at P83 and A177, demonstrating that residues near the pocket entrance play a critical role in guiding VD3 into a catalytically productive binding orientation. Specifically, A177M and A177P improved activity by 58 % and 53 %, respectively. Using A177M/K180F as the template for a second round of ISM, we obtained the triple variant P83A/A177M/K180F (L21‐M3), which showed a further 35 % increase in activity (Figure 7c). Kinetic characterization of purified L21‐M3 (Table 1) revealed improved substrate binding affinity (lower *K*_m_) and enhanced catalytic activity (higher *k*_cat_), resulting in a catalytic efficiency (*k*_cat_/*K*_m_) of 34.50 mM^−1^ s^−1^—a 3.2‐ fold improvement over L21. The coupling efficiency also improved from 24.42 % (in L21) to 36.34 % (in L21‐M3).

### 2.5 Computational Analysis of the Molecular Basis for VD3 Hydroxylation Catalyzed by L21 and L21‐M3

**FIGURE 8:**
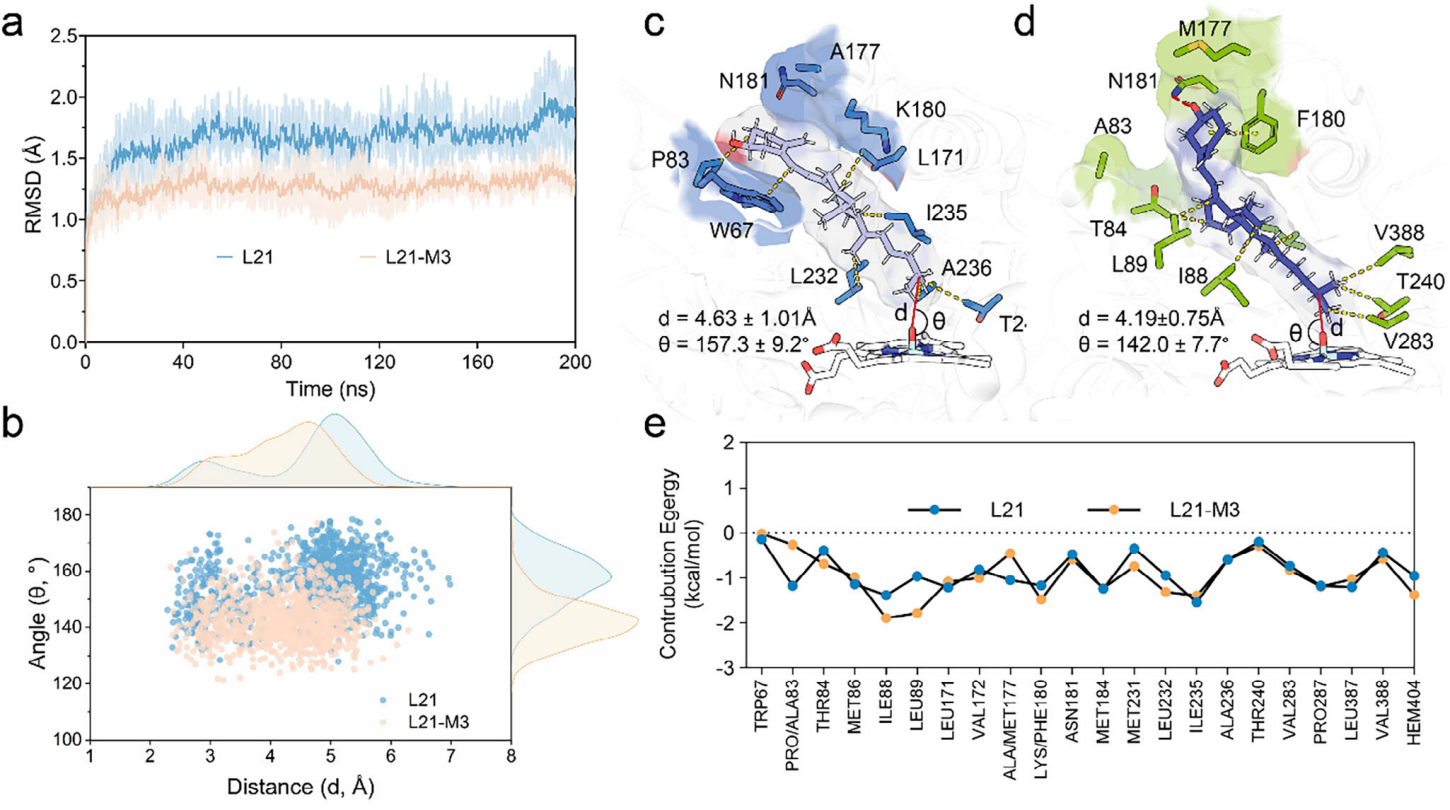
Mechanism analysis of VD3 hydroxylation by L21 and variant L21‐M3. (a) RMSD trajectories of L21 and variant L21‐M3 derived from three replicates of 200 ns MD simulations. (b) Statistical analysis of *d*[Fe═O─H] (d) and the angle between the C25 atom of VD3 and heme‐Fe═O (θ) derived from the 200 ns MD simulations. (c,d) Representative snapshots of the active site in (c) L21 and (d) L21‐M3. The yellow dashed line represents the hydrophobic interactions, and the red dashed line represents the H‐bond. (e) Per‐residue binding free energy contributions of residues surrounding VD3 in L21 and L21‐M3. *Note*: “d” stands for the *d*[Fe═O─H]; “θ” stands for the angle between the C25 atom of substrate and heme‐Fe═O. Mean values ± standard deviation are indicated above each distribution.

To elucidate the molecular mechanism underlying the enhanced activity and coupling efficiency of L21‐M3, MD simulations were employed to conduct comprehensive structural and computational analyses of the heme domains in both L21 and L21‐M3. A homology model of the L21‐M3 heme domain was generated using AlphaFold3. Each simulation system was constructed with VD3 bound, and the heme cofactor was replaced by Cpd I. RMSD trajectories confirmed that both systems reached equilibrium, with L21‐M3 exhibiting moderately enhanced overall stability (Figure 8a). Analysis of the *d*[Fe═O─H] distribution revealed that in L21, only 25 % of the configurations featured a *d*[Fe═O‐H] within 4 Å—a range conducive to Cpd I‐mediated catalysis [45]. In contrast, this proportion increased to 38 % in L21‐M3, accompanied by a decrease from 4.63 ± 1.01 Å (L21) to 4.19 ± 0.75Å (L21‐M3) (Figure 8b). Besides, the angle (θ) between the heme‐Fe═O group and the VD3 C25 atom decreased from 157.3 ± 9.2° (L21) to 142.0 ± 7.7° (L21‐M3), in agreement with previous research that the most favorable angle for hydroxidation is around 130°, which would reduce the activation barrier [45]. Thus, the smaller *d*[Fe═O─H] and the optimization of the angle (θ) between the heme‐Fe═O group and the C25 atom of VD3 increase the probability of productive H‐abstraction and reduce uncoupling side reactions, thus explaining the improved coupling efficiency and catalytic activity observed in L21‐M3.

Representative snapshots from the MD simulations were extracted based on the free energy landscape to examine mutation‐induced structural changes in the enzyme (Figure 8c,d). In the representative conformation of L21, VD3 was bound within the substrate pocket primarily via hydrophobic interactions. In L21‐M3, the mutation induced two key stabilizing interactions: (i) a hydrogen bond between N181 and the hydroxyl oxygen atom of VD3, and (ii) *π–π* stacking between the benzene ring of F180 (the K180F mutation) and the methylidenecyclohexan moiety of VD3. Additionally, M177 (the A177M mutation) functioned as a lid extending from the entrance of the substrate pocket, helping to anchor the head group of the substrate. Molecular mechanics/generalized born surface area (MM/GBSA) free energy calculations indicated no significant difference in the total binding free energy between L21‐M3 and VD3 compared to L21 (−47.74 ± 2.02 kcal·mol^−^^1^ to ‒49.17 ± 3.96 kcal·mol^−^^1^; Table S3). However, enhanced binding energy contributions were observed from residues T84, I88, L89, K180, M231, L232, V283, and V388 in L21‐M3 (Figure 8e). These structural alterations promote deeper insertion of VD3 into the binding pocket, inducing a conformational twist in the substrate and reducing *d*[Fe═O─H]. Consequently, the hydrophobic interactions between the terminal propyl group of VD3 and the pocket are strengthened, restricting the flexibility of the propyl tail and stabilizing a catalytically productive pose.

### 2.6 Semi‐Preparative‐Scale Production of 25(OH)VD3

**FIGURE 9:**
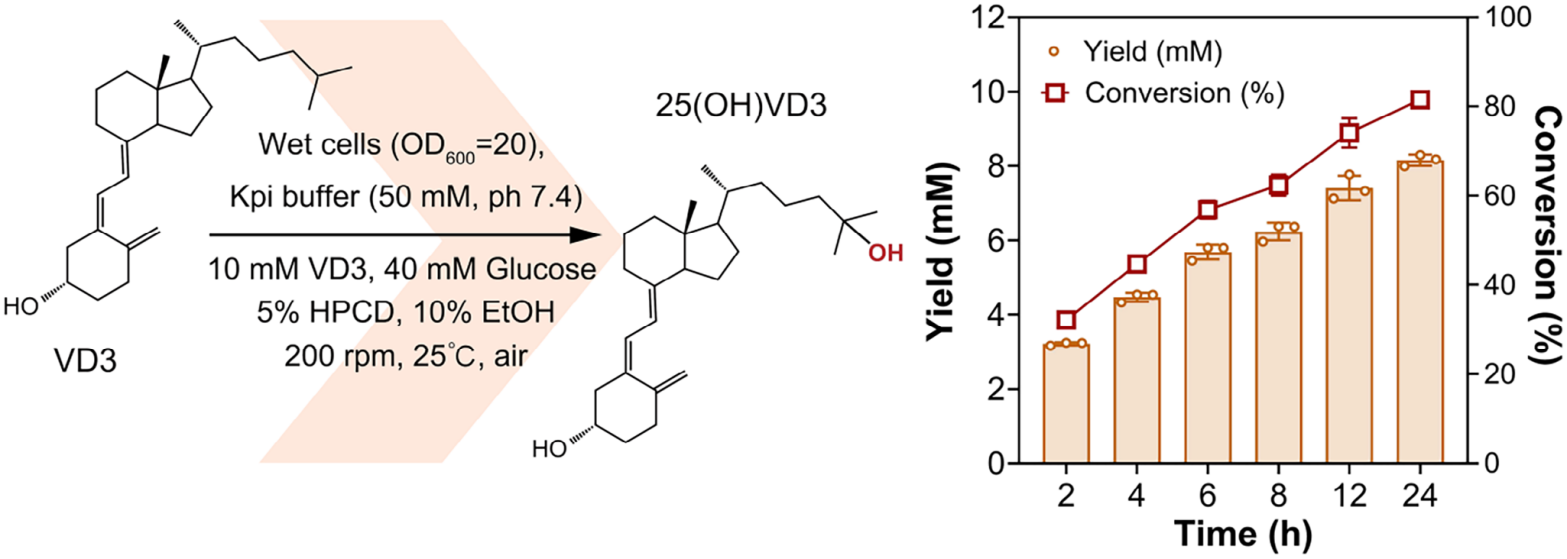
Semi‐preparative‐scale production of 25(OH)VD3. Reaction conditions: recombinant *E. coli* cells frozen at ‒80°C were resuspended in potassium phosphate buffer (50 mM, pH 7.4) to an OD_600_ of 20, containing 10 mM VD3, 40 mM glucose, 5 % (w/v) HPCD, and 10 % (v/v) ethanol as a cosolvent. The reaction was carried out at 25°C with shaking at 200 rpm for 24 h.

We subsequently combined the beneficial mutations from L21‐M2 (ET pathway optimization) and L21‐M3 (heme domain/substrate binding optimization) to construct a quintuple variant, designated P83A/A177M/K180F/F346K/R354M (L21‐M5). In this variant, the ET pathway, redox‐domain dynamics, and substrate specificity were concurrently optimized. Compared to L21, L21‐M5 exhibited an 8.2‐ fold improvement in catalytic efficiency (75.92 mM^−1^ s^−1^), a 3‐ fold higher TTN, and a coupling efficiency of 56.78 %—representing a 33.59 % increase relative to L21 (Table 1). We then employed an *E. coli* BL21(DE3) strain co‐expressing L21‐M5 and GDH to construct a whole‐cell biocatalyst for the semi‐preparative‐scale synthesis of 25(OH)VD3. Following previously established procedures, whole‐cell catalysis was performed at a cell density OD_600_ of 20 with 10 mM VD3 as the substrate (Figure 9). After 24 h of reaction, the product concentration reached 8.16 mM (3.26 g/L), corresponding to a conversion rate of 82 %. The product was confirmed by nuclear magnetic resonance (NMR) spectroscopy (Figure S13). This productivity underscores the effectiveness of our comprehensive ET optimization strategy for self‐sufficient cytochrome P450 enzymes and highlights its potential for industrial applications.

## 3 Conclusions

Here, we developed an ET optimization strategy integrating conformational dynamics modulation, ET pathway engineering, and substrate orientation tuning to enhance ET efficiency in self‐sufficient cytochrome P450s for 25(OH)VD3 biosynthesis. By optimizing conformational dynamics, [2Fe‐2S]→heme ET pathways, and heme domain substrate pockets, we addressed key ET bottlenecks of P450s. The quintuple variant L21‐M5 (combining beneficial mutations) showed an 8.2‐ fold higher catalytic efficiency, 3‐ fold increased total turnover number, and 56.78 % coupling efficiency. An *E. coli* strain expressing L21‐M5 produced 3.26 g/L 25(OH)VD3 in 24 h with 82% conversion. This framework advances P450 engineering beyond localized optimization, offering a versatile tool for multi‐domain redox enzyme enhancement and the green synthesis of pharmaceuticals and natural products, and chiral fine chemicals.

## 4 Experimental Section/Methods

### 4.1 Materials and Strains

VD3, 25(OH)VD3, 2‐hydroxypropyl‐β‐cyclodextrin (HPCD), isopropyl β‐d‐1‐thiogalactopyranoside (IPTG), and 5‐aminolevulinic acid (5‐ALA) were purchased from Aladin (Shanghai, China). All enzymes and buffers required for cloning were obtained from Vazyme Biotech (Nanjing, China). All other chemicals were sourced from reputable suppliers and were of the highest available purity. The *E. coli* Trans‐1 and *E. coli* BL21 (DE3) were employed as hosts for gene cloning and protein expression, respectively.

### 4.2 Construction of Recombinant Whole‐Cell Biocatalyst

Codon optimization was first conducted on the genes encoding P450s and glucose dehydrogenase (GDH; PDB ID: 3AUS). These optimized genes were then synthesized by Sangon Biotech (Shanghai, China) and subsequently cloned into the expression vector pETDuet‐1 through homologous recombination [49]. When constructing multicistronic vectors, a synthetic ribosome‐binding site (RBS; sequence: 5’‐GAGCTCGGTACCGGGGATCC‐3’) was inserted between two adjacent coding sequences [35]. Site‐directed mutagenesis and combinatorial mutagenesis were carried out via polymerase chain reaction (PCR)‐mediated approaches, with the Phusion polymerase kit employed for the experimental procedures. Recombinant plasmids that had passed verification were introduced into competent *E. coli* BL21(DE3) cells, which were used to support protein expression and the development of whole‐cell biocatalysts.

### 4.3 Preparation of Whole‐Cell Catalysts

Recombinant *E. coli* BL21(DE3) cells harboring engineered plasmids were pre‐cultured in 40 mL of Luria–Bertani (LB) medium (50 µg/mL ampicillin) at 37°C for 6–8 h with shaking at 200 rpm. A 4 mL aliquot of the pre‐culture was inoculated into 400 mL of Terrific Broth (TB) medium supplemented with 50 µg/mL ampicillin and incubated at 37°C, 200 rpm, until the OD_600_ reached 0.6∼0.8. Thereafter, protein expression was triggered through the addition of 0.5 mM IPTG and 0.5 mM 5‐ALA, after which incubation was continued at 25°C for 18 h. Cells were harvested via centrifugation (6000 ×g, 25°C, 5 min), subjected to two washes with 50 mM potassium phosphate buffer (PB, pH 7.4), and stored at −80°C for use in subsequent biotransformation experiments.

### 4.4 Activity Assay of P450s Whole‐Cell Biocatalysts

Recombinant *E. coli* cells were suspended in 50 mM potassium phosphate buffer (pH 7.4, OD_600_ = 10) for the assay, with the buffer supplemented with 10 mM glucose, 5 % (w/v) hydroxypropyl‐β‐cyclodextrin (HPCD), and 10 % (v/v) ethanol (used as a cosolvent). Biocatalytic reactions were started via the addition of 1 mM VD3 (from a 200 mM stock solution prepared in ethanol) into 10 mL of the cell suspension placed in 100 mL Erlenmeyer flasks. Reactions proceeded at 25°C under shaking at 200 rpm. At specified time points, 500 µL aliquots were collected; each aliquot was mixed with an equal volume of ethyl acetate and vortexed for 1 min to stop the reaction. Following phase separation via centrifugation (12 000 ×*g*, 5 min), the organic phase was harvested and filtered through a 0.22 µm nylon membrane—this filtered phase was used for subsequent High‐Performance Liquid Chromatography (HPLC) analysis. The substrate conversion rate (%) is calculated according to the following equation based on HPLC: Conversion (%) = M_product_ / (M_product_+M_substrate_) × 100.

### 4.5 HPLC and NMR Analysis

The products of VD3 biotransformation were analyzed using a Shimadzu LC‐20AD system equipped with an Agilent C18 (250 × 4.6 mm, 5 µm) column. The mobile phase consisted of methanol/acetonitrile (30:70, v/v), with a detection wavelength of 264 nm, a flow rate of 1.0 mL/min, an injection volume of 10 µL, and a column temperature of 30°C [50]. The structures of the prepared 25(OH)VD3 were confirmed by ^1^H. The NMR spectra were acquired on a Bruker AVANCE III 600 MHz spectrometer at 25°C. The spectra were processed using MestReNova 14.0.

### 4.6 Measurement of Kinetic Parameters and Coupling Efficiency

Recombinant *E. coli* cells were reconstituted in ice‐cold 50 mM potassium phosphate buffer (pH 7.4) for subsequent processing, then disrupted via ultrasonication for 30 min in an ice‐water bath. Following centrifugation (12 000 ×*g*, 4°C, 30 min), the supernatant was subjected to purification using Ni‐NTA affinity chromatography. Protein concentration was quantified via the Bradford assay. For kinetic parameter determination, reactions (final volume: 0.5 mL) were performed in 50 mM potassium phosphate buffer (pH 7.4) containing 1 µM purified P450, substrate concentrations ranging from 0.01–5 mM, and 5 mM NADH at 25°C and 1000 rpm for 10 min [51]. The Michaelis–Menten equation was used to calculate the *K*_m_ and *V*_max_ values using GraphPad Prism 8.0. For total turnover number (TTN) measurements, 0.5 mL reactions containing purified 0.2 µM P450s in 50 mM potassium phosphate buffer (pH 7.4), 1 mM VD3, and 5 mM NADH were conducted at 25°C and 200 rpm for 4 h. For coupling efficiency measurements, reactions (0.5 mL) containing 1 µM purified P450s, 1 mM VD3, and 1 mM NADH were conducted at 25°C and 1000 rpm until complete consumption of NADH [25]. The products were extracted with 500 µL ethyl acetate, and the organic phase was filtered through a 0.22 µm nylon membrane for HPLC analysis. The TTN and coupling efficiency (%) are calculated according to the following equations:TTN = product concentration/P450 concentration; Coupling efficiency = (the content of product / the content of NADH consumption) ×100.

### 4.7 Prediction and Analysis of the Structures of P450s

The crystal structures of CYP116B46 (PDB ID: 6LAA) and Vdh‐K1 with bound vitamin D3 (PDB ID: 3A50) were available from the website RCSB Protein Data Bank (https://www.rcsb.org/). Structures of full‐length VK1‐CYP116B46‐L13 (L13), VK1‐CYP116B46‐L21 (L21), VK1‐CYP116B46‐L21‐F346K/R354M (L21‐M2) and the heme domain of VK1‐CYP116B46‐L21‐P83A/A177M/K180F (L21‐M3) were modeled using AlphaFold3 [40]. FMN and the experimental substrate VD3 were docked into their respective domain using Smina [43]. Using PyMOL, the structures of VK1‐CYP116B46‐L21 and CYP116B46 were superimposed to obtain their aligned conformation.

### 4.8 Computational System Setup and Molecular Dynamics Simulations

Five protein systems—L13, L21, L21‐M2, the heme domain of L21, and the heme domain of L21‐M3—were prepared for computational analysis. In L13, L21, and L21‐M2 systems, the [2Fe‐2S] cluster was modeled in the one‐electron‐reduced state, with one iron atom in the ferric state and the other in the ferrous state, while the heme iron remained in the oxidized ferric state. The [2Fe‐2S] cluster in the ferredoxin domain and the heme moiety were parametrized using the “MCPB.py” tool [52, 53] of AmberTools18 [54]. The parameters for the high‐spin (S = 5/2) oxidized heme were adopted from previously published literature [55]. The general AMBER force field (GAFF) [56] was used to generate the parameters for FMN and the VD3, with the partial atomic charges derived using the RESP method [57] at the HF/6‐31G* level of theory. Missing parameters for the substrates and co‐substrates were supplemented using the parmchk2 utility in AmberTools18 [54]. For the L21 and L21‐M3 heme‐domain systems, parameters for the CpdI heme and its axial cysteine were taken from Ref. [34]; all other parameters remained unchanged. The Amber ff14SB force field [58] was employed for the normal protein residues. Protonation states of titratable residues (histidine, glutamic acid, and aspartic acid) were assigned based on predicted pKa values from the PROPKA [59] combined with manual inspection of local hydrogen‐bonding networks. Histidine residues at positions 11, 23, 69, 95, 188, 238, 255, 342, 345, 430, 447, 491, 514, 547, 566, 571, 624, 631, and 693 were protonated at the ε‐nitrogen position. All glutamic acid and aspartic acid residues were modeled in their deprotonated (anionic) forms. Sodium ions were added to neutralize the overall charge of each system. The resulting protein‐ligand complexes were then solvated in a rectangular TIP3P water box, with a minimum distance of 12 Å between the protein surface and the edge of the box.

### 4.9 Molecular Dynamics Simulations

After completing system preparation, energy minimization was carried out using a combination of the steepest descent and conjugate gradient algorithms. The systems were then gradually heated from 0 to 300 K over 50 ps under the canonical (NVT) ensemble, applying a weak positional restraint of 25 kcal·mol^−^^1^·Å^−^^2^ on the protein backbone atoms. This was followed by a 1 ns equilibration under the isothermal‐isobaric (NPT) ensemble at 300 K and 1.0 atm, allowing the system to reach a uniform density. Subsequently, all restraints were removed, and the systems were further equilibrated for 4 ns. Production molecular dynamics (MD) simulations were then performed for 200 ns for each system. Three independent replicates were conducted to ensure reproducibility. Throughout all MD simulations, covalent bonds involving hydrogen atoms were constrained using the SHAKE algorithm [60], and a 2 fs integration time step was employed. All simulations were performed using the GPU‐accelerated version of the AMBER18 software package [54]. The resulting trajectories were analyzed by using the CPPTRAJ module in AmberTools23 [61].

### 4.10 Umbrella Sampling Simulation and Potential of Mean Force Calculation

The umbrella sampling [62] combined with restraint MD simulations in AMBER was used to explore the “proximal” conformation of the [2Fe–2S] cluster. An initial “distal” conformation was obtained via a 200 ns conventional MD simulation after the equilibration of the starting structure. The distance between one of the Fe atoms of the [2Fe‐2S] cluster and the heme‐Fe atom (we denote it as *d*[FeS‐Heme] in the following paragraphs) was chosen as the reaction coordinate (RC). Consistent with earlier studies on related self‐sufficient P450 systems [19], conformations with *d*[FeS–Heme] > 22 Å were classified as distal, whereas those with *d*[FeS–Heme] < 20 Å were classified as proximal. The “proximal” conformation was subsequently sampled by performing a 200 ns conventional MD simulation on structures extracted from the umbrella sampling trajectory. For L21‐M2, 46 windows were constructed to cover RC ranging from 14.1 to 23.1 Å with an interval of 0.2 Å for two adjacent windows, and the force constant was set to be 100 kcal·mol^−1^ Å^−2^. For L13, RC ranges from 14.1 to 27.9, and for L21, RC ranges from 14.1 to 25.9. The same interval and force constant were used for the umbrella samplings of L13 and L21. For each window, a 100 ns MD simulation with a biasing harmonic potential was carried out. During all restraint MD simulations, the covalent bonds containing hydrogen were constrained using SHAKE, and an integration step of 1 fs was used. PMF was constructed with the weighted histogram analysis method (WHAM)/multistate Bennett acceptance ratio method (MBAR) using the RC data in the last 85 ns restraint production simulations of each window. Since the [2Fe‐2S] cluster is located at a flexible loop, the “distal‐to‐proximal” transition involves collective motions of many degrees of freedom, *d*[FeS‐Heme] may not be an optimal RC to distinguish between the “proximal” and the “distal” conformation. Therefore, principal component analysis was performed on the backbone dihedrals, then the first principal component (PC1) was set as a new RC to which the potential of mean force (PMF) was projected. The projecting procedure was performed with the pymbar module [63]. Here, we briefly describe the projecting procedure: First, the free energies of each biased ensemble were calculated from the MBAR equation (Equation 1), and the weights of each frame used for PMF construction were obtained (Equation 2). Then the probability density and PMF as a function of new RC were calculated by reweighing using the weights and RC values (Equation 3). Finally, the PMF was calculated with Equation 4.

(1) $$\omega_{0}\;\left(r\right)=\frac{e^{-\beta U_{0}\left(r\right)}}{\sum_{k=1}^{K}N_{k}e^{-\beta \left(U\left(r\right)-f_{k^{\prime }}\right)}}\;$$

(2)

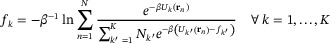

(3) $$P_{0}\left(s_{m}\right)=\frac{\sum_{n=1}^{N}\omega_{t}\left(r_{n}\right)\delta \left(s_{m}-s\left(r_{n}\right)\right)}{\sum_{n=1}^{N}\omega_{t}\left(r_{n}\right)}\;$$

(4) $$F_{0}\left(s\right)=-k_{B}T\ln\left[P_{0}\left(s\right)\right]$$

### 4.11 Calculation of Electron Transfer Efficiency

In enzymatic reactions, the electron transfer rate (*k*_ET_) [64, 65] can be simplified to a distance‐dependent equation:

(5) $$k_{\mathrm{ET}}=A\left(0\right)\;e^{-\beta R}$$

where *β* is a measure of the ability to couple or superexchange electrons; R is the distance between the electron donor and the electron acceptor; and *k*_ET_ characterizes the rate of transferring electrons in units of S^−1^. According to Ivanov et al. [66], this *k*_ET_ was calculated with the values of A(0) = 2 × 10^12^ and *β* = 1.06 Å in this study. R was calculated by adding the shortest distances between neighboring residues in the shortest pathway of electron transfer.

### 4.12 Molecular Mechanics / Generalized Born Surface Area (MM/GBSA) Free Energy Calculations Process

The last 50 ns trajectories derived from the MD simulations were analyzed by using MM/GBSA calculations for VD3 in complexes L21 and L21‐M3. The default dielectric constant value (ε = 1) and the salt concentration of 0.15 M were set. Frames used for these calculations were extracted every 100 ps during the 50 ns MD simulations of the complexes.

### 4.13 Statistical Analysis

Three biological replicates were conducted to determine statistical values (mean ± standard deviation). GraphPad Prism 10.0 was used for graphing and statistical analyses. Pearson's correlation analysis in the experimental group was performed. Statistical significance was set at *p* < 0.05.

## Conflicts of Interest

The authors declare no conflict of interest.

## Supporting information




**Supporting File**: advs73900‐sup‐0001‐SuppMat.docx.

## Data Availability

The data that support the findings of this study are available from the corresponding author upon reasonable request.
