# Supplementary material for: Synergistic Tuning of Conformational Dynamics, Electron Tunneling, and Substrate Positioning Enhances Electron Transfer in a P450 Chimera for Calcifediol Biosynthesis
Source: Adv Sci (Weinh). 2026 Jan 20;13(22):e19381. doi: 10.1002/advs.202519381 (PMC13088337; doi:10.1002/advs.202519381)
Supplement: Supplementary file 1 — Supporting File: advs73900‐sup‐0001‐SuppMat.docx. [file ADVS-13-e19381-s001.docx]

**Supplementary information**

**Synergistic Tuning of Conformational Dynamics, Electron Tunneling, and Substrate Positioning Enhances Electron Transfer in a P450 Chimera for Calcifediol Biosynthesis**

*Ziqi Liang, Xitong Song, Shuming Cheng, Yiwen Shen, Jie Zhang, Yaru Wang, Huiying Luo, Bin Yao, Binju Wang* and Tao Tu**

Z Liang, Y Shen, J Zhang, Y Wang, H Luo, B Yao, T Tu

State Key Laboratory of Animal Nutrition and Feeding, Institute of Animal Sciences, Chinese Academy of Agricultural Sciences, Beijing 100193, China

E-mail: [tutao@caas.cn](mailto:tutao@caas.cn)

Z Liang

College of Animal Science and Technology, Northwest A&F University, Yangling 712100, Shaanxi, China

S Cheng, B Wang

State Key Laboratory of Physical Chemistry of Solid Surfaces and Fujian Provincial Key Laboratory of Theoretical and Computational Chemistry, College of Chemistry and Chemical Engineering, Xiamen University

361005 Xiamen, P. R. China

E-mail: [wangbinju2018@xmu.edu.cn](mailto:wangbinju2018@xmu.edu.cn)

X Song

Fujian Provincial Key Laboratory of Ecological Impacts and Treatment Technologies for Emerging Contaminants, College of Environmental and Biological Engineering, Putian University, Putian 351100, China.

**Supplementary Tables**

**Table S1.** The amino acid sequence of the entire fusion protein of P450s.

| P450s | Amino acid sequence of fusion protein |
| --- | --- |
| VK1-CYP116B46-L13 | MALTTTGTEQHDLFSGTFWQNPHPAYAALRAEDPVRKLALPDGPVWLLTRYADVREAFVDPRLSKDWRHTLPEDQRADMPATPTPMMILMDPPDHTRLRKLVGRSFTVRRMNELEPRITEIADGLLAGLPTDGPVDLMREYAFQIPVQVICELLGVPAEDRDDFSAWSSVLVDDSPADDKNAAMGKLHGYLSDLLERKRTEPDDALLSSLLAVSDEDGDRLSQEELVAMAMLLLIAGHETTVNLIGNGVLALLTHPDQRKLLAEDPSLISSAVEEFLRFDSPVSQAPIRFTAEDVTYSGVTIPAGEMVMLGLAAANRDADWMPEPDRLDITRDASGGVFFGHGIHFCLGAQLARLEGRVAIGRLFADRPELALAVGLDELVYRESTLVRGLSRMPVTMGPRSALRQPVRIGPPRAKDVVRTMEVAAVERPSEDIVVLHLTRPDRRPLPRWSPGAHIDIECGEPDRSRQYSLCSDPENRDAWRVAVQRDPASRGGSRWIHEEVRPGMLLRVRGPRNSFRLDEHAPRYLFLAGGIGITPIMTMAARAKELGTDYELHYSVRSRTSLIFVDELRQIHGDRLHVYVSEEGVRNDLAALIRRASAGTQIYACGPQRMLDTLERLIENRPEVTLRVEHFFGEPSHLDPAKERPFQVVLRNSGLTVEVPADKTLLEVLRAYNIEVQSDCEEGLCGTCEVSVVEGEVDHRDSVLTRAERRENRRMMCCCSRAKTERLVLDL |
| VK1-CYP116B46-L21 | MALTTTGTEQHDLFSGTFWQNPHPAYAALRAEDPVRKLALPDGPVWLLTRYADVREAFVDPRLSKDWRHRLPEDQRADMPATPTPMMILMDPPDHTRLRKLVGRSFTVRRMNELEPRITEIADGLLAGLPTDGPVDLMREYAFQIPVQVICELLGLPAEDRDDFSAWSSVLVDDSPADDKNAAMGKLHGYLSDLLERKRTEPDDALLSSLLAVSDMDGDRLSQEELVAMAMLLLIAGHETTVNLIGNGVLALLTHPDQRKLLAEDPSLISSAVEEFLRFDSPVSQAPIRFTAEDVTYSGVTIPAGEMVMLGLAAANRDADWMPEPDRLDITRDASGGVFFGHGIHFCLGAQLARLEGRVAIGRLFADRPELALAVGLDELVYRRSTLVRGLSRMPVTMGPRSAPPRAKDVVRTMEVAAVERPSEDIVVLHLTRPDRRPLPRWSPGAHIDIECGEPDRSRQYSLCSDPENRDAWRVAVQRDPASRGGSRWIHEEVRPGMLLRVRGPRNSFRLDEHAPRYLFLAGGIGITPIMTMAARAKELGTDYELHYSVRSRTSLIFVDELRQIHGDRLHVYVSEEGVRNDLAALIRRASAGTQIYACGPQRMLDTLERLIENRPEVTLRVEHFFGEPSHLDPAKERPFQVVLRNSGLTVEVPADKTLLEVLRAYNIEVQSDCEEGLCGTCEVSVVEGEVDHRDSVLTRAERRENRRMMCCCSRAKTERLVLDL |

The Vdh-K1 sequence is highlighted in green, the linker sequence in red, and the reductase domain of CYP116B46 in blue.

**Table S2.** Primers used in this study.

| Primer name | Sequence (5’ to 3’) |
| --- | --- |
| F340X-F | GGCGTGTTC**NNK**GGTCATGGTATCCAT |
| F340X-R | ATACCATGACCGAA**MNN**CACGCCACCGCTC |
| F346X-F | TGGTATCCAT**NNK**TGCCTGGGTGCACAG |
| F346X-R | CCAGGCA**MNN**ATGGATACCATGACCGAAGAAC |
| R354X-F | ACAGCTGGCA**NNK**CTGGAAGGTCGTGTT |
| R354X-R | GACCTTCCAG**MNN**TGCCAGCTGTGCACC |
| R664X-F | GGAAGTTTTG**NNK**GCATACAACATCGAA |
| R664X-R | TGTTGTATGC**MNN**CAAAACTTCCAACAG |
| I668X-F | TGCATACAAC**NNK**GAAGTGCAGTCTGAT |
| I668X-R | GCACTTC**MNN**GTTGTATGCACGCAAAACTTCC |
| E669X-F | CAACATC**NNK**GTGCAGTCTGATTGTGAAGAAGGT |
| E669X-R | ACTGCAC**MNN**GATGTTGTATGCACGCAAAACTTC |
| V670X-F | CATCGAA**NNK**CAGTCTGATTGTGAAGAAGGTCTGTG |
| V670X-R | CAGACTG**MNN**TTCGATGTTGTATGCACGCAAA |
| Q671X-F | CATCGAAGTG**NNK**TCTGATTGTGAAGAAGGTCTGTGC |
| Q671X-R | CAGA**MNN**CACTTCGATGTTGTATGCACGCAAA |
| S672X-F | AGTGCAG**NNK**GATTGTGAAGAAGGTCTGTGCGG |
| S672X-R | CACAATC**MNN**CTGCACTTCGATGTTGTATGCAC |
| D673X-F | GCAGTCT**NNK**TGTGAAGAAGGTCTGTGCGGCA |
| D673X-R | CTTCACA**MNN**AGACTGCACTTCGATGTTGTATGC |
| E675X-F | TGT**NNK**GAAGGTCTGTGCGGCACTTGTGAAGT |
| E675X-R | CACAGACCTTC**MNN**ACAATCAGACTGCACTTCGATGTT |
| T681X-F | TGTGCGGC**NNK**TGTGAAGTTTCTGTTGTTGAAGGTG |
| T681X-R | TTCACA**MNN**GCCGCACAGACCTTCTTCACAAT |
| P83X-F | AACC**NNK**ACCCCGATGATGATCCTGATGGACC |
| P83X-R | TCATCGGGGT**MNN**GGTTGCCGGCATATCAGCG |
| M86X-F | ACCCCG**NNK**ATGATCCTGATGGACCCGCCGGA |
| M86X-R | AGGATCAT**MNN**CGGGGTCGGGGTTGCCGGCAT |
| I88X-F | GATGATG**NNK**CTGATGGACCCGCCGGATCACA |
| I88X-R | CCATCAG**MNN**CATCATCGGGGTCGGGGTTGCC |
| L89X-F | GATGATC**NNK**ATGGACCCGCCGGATCACACCC |
| L89X-R | GGTCCAT**MNN**GATCATCATCGGGGTCGGGGTT |
| L171X-F | GTGGAGTTCTGTT**NNK**GTTGATGATTCTCCGGCTGATG |
| L171X-R | C**MNN**AACAGAACTCCACGCAGAGAAATCATCA |
| V172X-F | A**NNK**GATGATTCTCCGGCTGATGATAAAAACG |
| V172X-R | CCGGAGAATCATC**MNN**TAAAACAGAACTCCACGCAGAGAA |
| A177X-F | GATTCTCCG**NNK**GATGATAAAAACGCGGCTATGG |
| A177X-R | TCATC**MNN**CGGAGAATCATCAACTAAAACAGAA |
| K180X-F | TGATGAT**NNK**AACGCGGCTATGGGTAAACTGC |
| K180X-R | CCGCGTT**MNN**ATCATCAGCCGGAGAATCATCA |
| N181X-F | GATGATAAA**NNK**GCGGCTATGGGTAAACTGCA |
| Primer name | Sequence (5’ to 3’) |
| N181X-R | GCCGC**MNN**TTTATCATCAGCCGGAGAATCATC |
| M184X-F | ACGCGGCT**NNK**GGTAAACTGCACGGTTACCTGTC |
| M184X-R | TTTACC**MNN**AGCCGCGTTTTTATCATCAGCCG |
| M231X-F | TTGCTATGGCC**NNK**CTGCTGCTGATTGCGGGC |
| M231X-R | CAG**MNN**GGCCATAGCAACCAGTTCTTCCTGAG |
| T240X-F | GCCATGAA**NNK**ACTGTGAACCTGATCGGCAACG |
| T240X-R | CACAGT**MNN**TTCATGGCCCGCAATCAGCAGCA |
| V283X-F | TTCGACTCTCCG**NNK**AGCCAGGCTCCGATCCGT |
| V283X-R | CT**MNN**CGGAGAGTCGAAACGCAGGAATTCTTC |
| P287X-F | CCAGGCT**NNK**ATCCGTTTCACCGCGGAAGATG |
| P287X-R | AACGGAT**MNN**AGCCTGGCTAACCGGAGAGTCG |
| L387X-F | AAGCACC**NNK**GTTCGTGGTCTGAGCCGCATGC |
| L387X-R | CACGAAC**MNN**GGTGCTTTCACGGTAAACCAGT |
| K180F/A177X-F | GATTCTCCG**NNK**GATGATTTTAACGCGGCTATGG |
| K180F/A177X-R | TCATC**MNN**CGGAGAATCATCAACTAAAACAGAA |

**Table S3.** Binding free energies of L21 and L21-M3 with VD3 by MM/GBSA.

| System | L21 | L21-M3 |
| --- | --- | --- |
| ΔE*_vdW_* | -50.35±1.79 | -51.05±3.24 |
| ΔE*_ele_* | -0.86±1.37 | -3.35±3.46 |
| ΔG*_GB_* | 9.84±0.99 | 11.46±3.26 |
| ΔE*_nonpolar_* | -6.37±0.29 | -6.23±0.35 |
| ΔG*_gas_* | -51.21±1.86 | -54.4±6.5 |
| ΔG*_solv_* | 3.47±1.00 | 5.23±3.05 |
| ΔG*_total_* | -47.74±2.02 | -49.17±3.96 |

**Supplementary Figures**


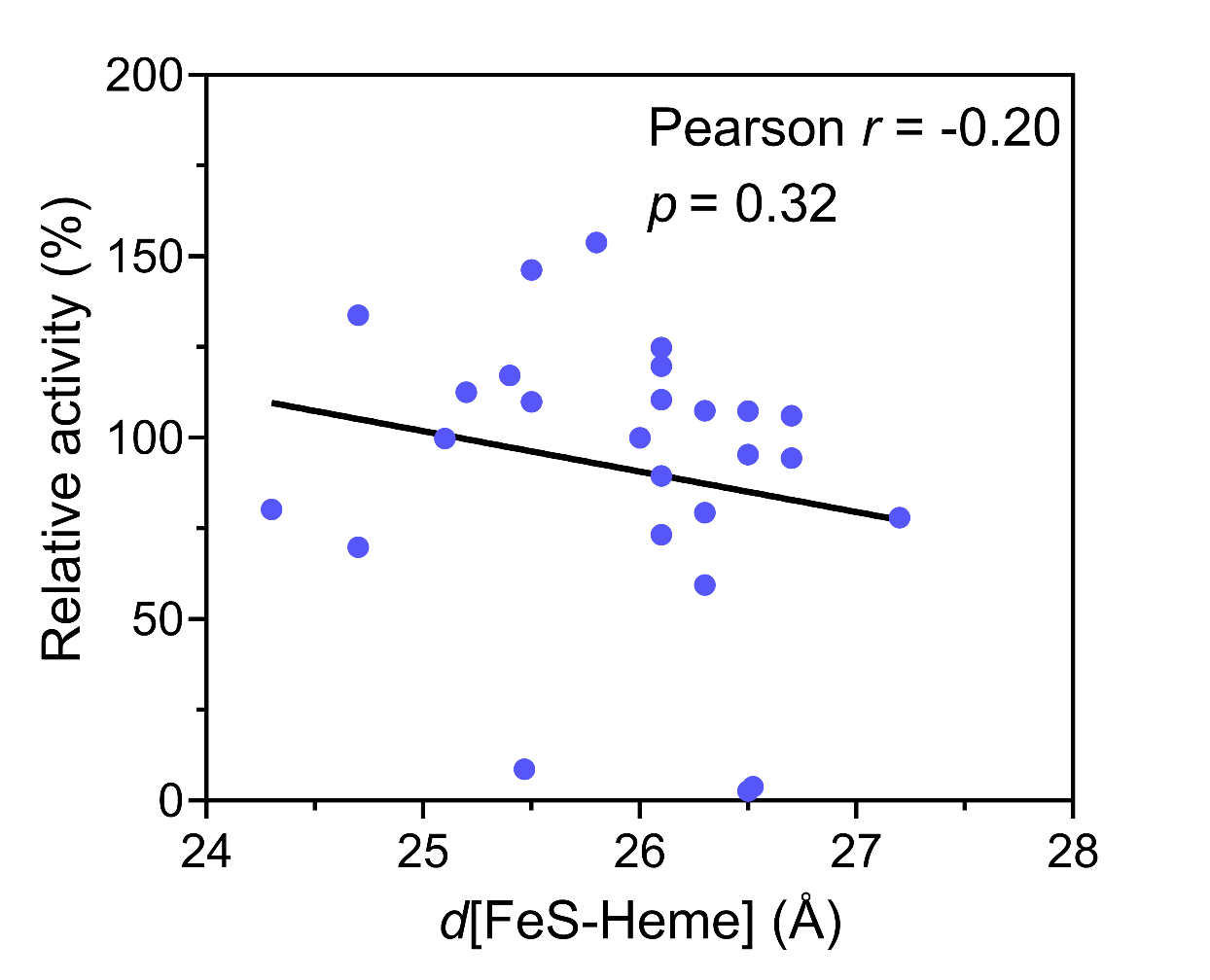


**Figure S1.** Pearson correlation analysis between the relative activity and the d[FeS–heme]. No significant correlation was observed between the catalytic activity and the d[FeS–heme] across the 26 chimeric P450 constructs (Pearson r = –0.20, *p* = 0.32). Significance levels were defined as *p* < 0.05.


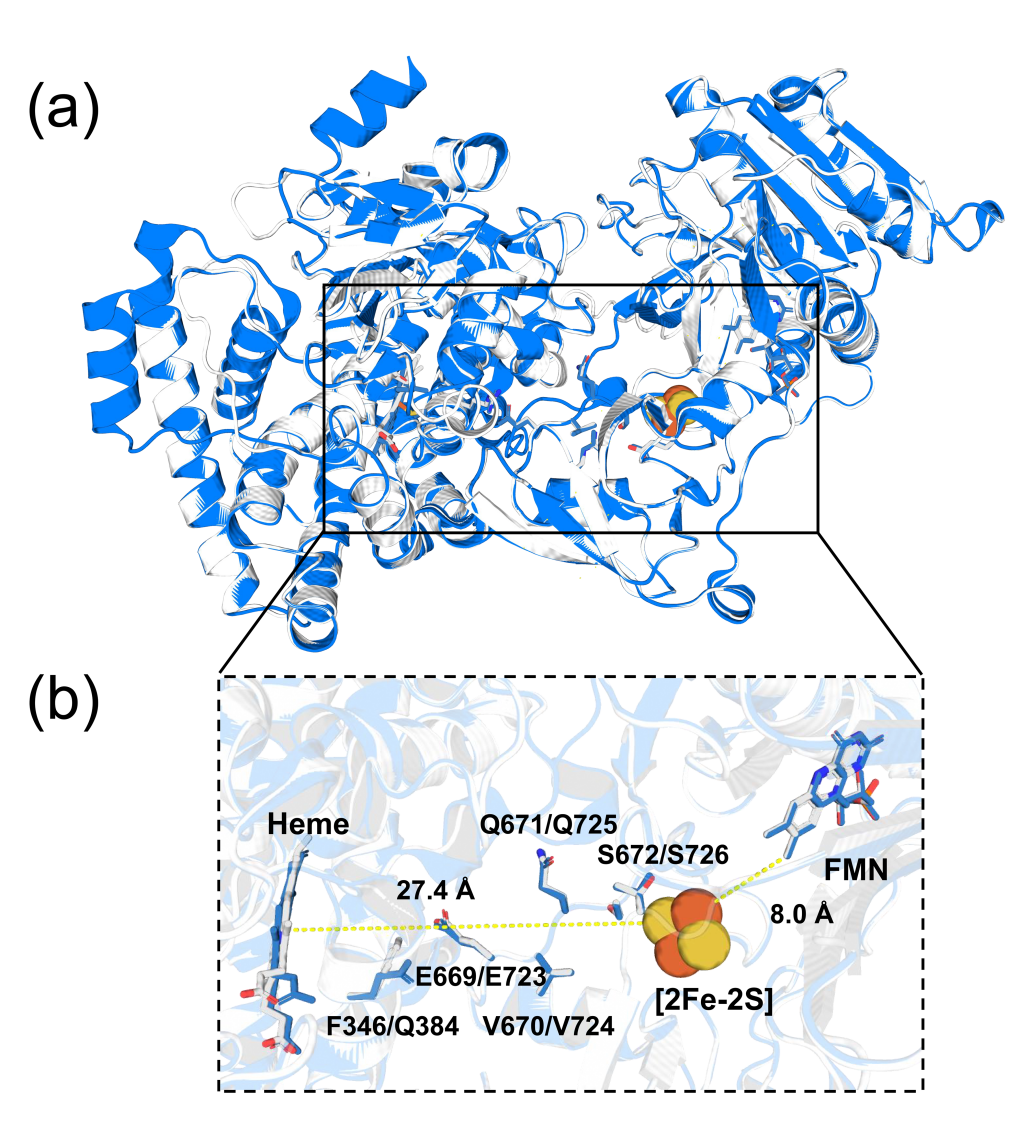


**Figure S2.** (a) Superimposed overall structures of VK1-CYP116B46-L21 (white) and CYP116B46 (blue). Root-mean-square deviation (RMSD) = 0.121 Å. (b) Residues involved in the [2Fe-2S]→heme ET pathway ET pathway in CYP116B46 identified by the “Pathways” plugin and the equivalent positions in L21. The FMN–[2Fe-2S] distance (*d*[FMN-FeS]) and the [2Fe-2S]–heme distance (*d*[FeS-Heme]) in L21 were 27.4 Å and 8.0 Å, respectively.


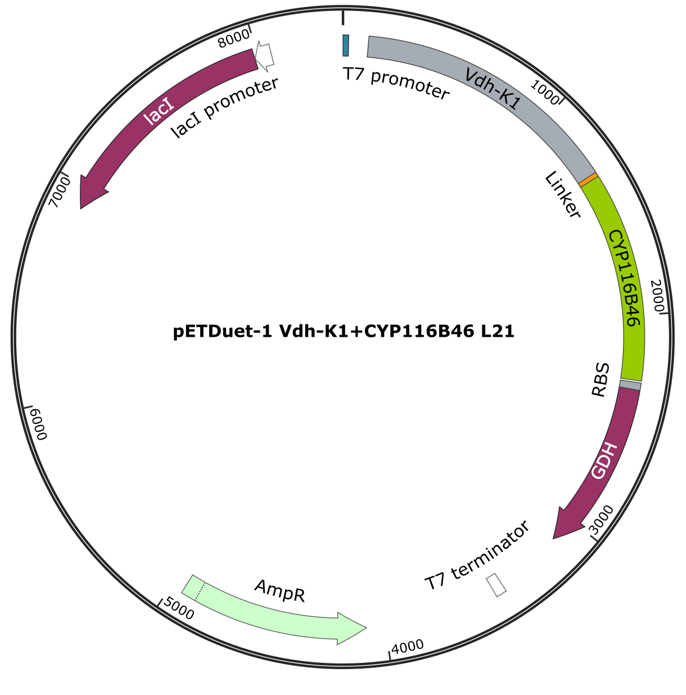


**Figure S3.** Configurations of recombinant plasmids used for chimeric P450s activity test. The genes of the chimeric P450s were inserted into pETDuet-1 with GDH. The RBS sequence (GAGCTCGGTACCGGGGATCC, blue bar) was inserted between chimeric P450s and GDH.


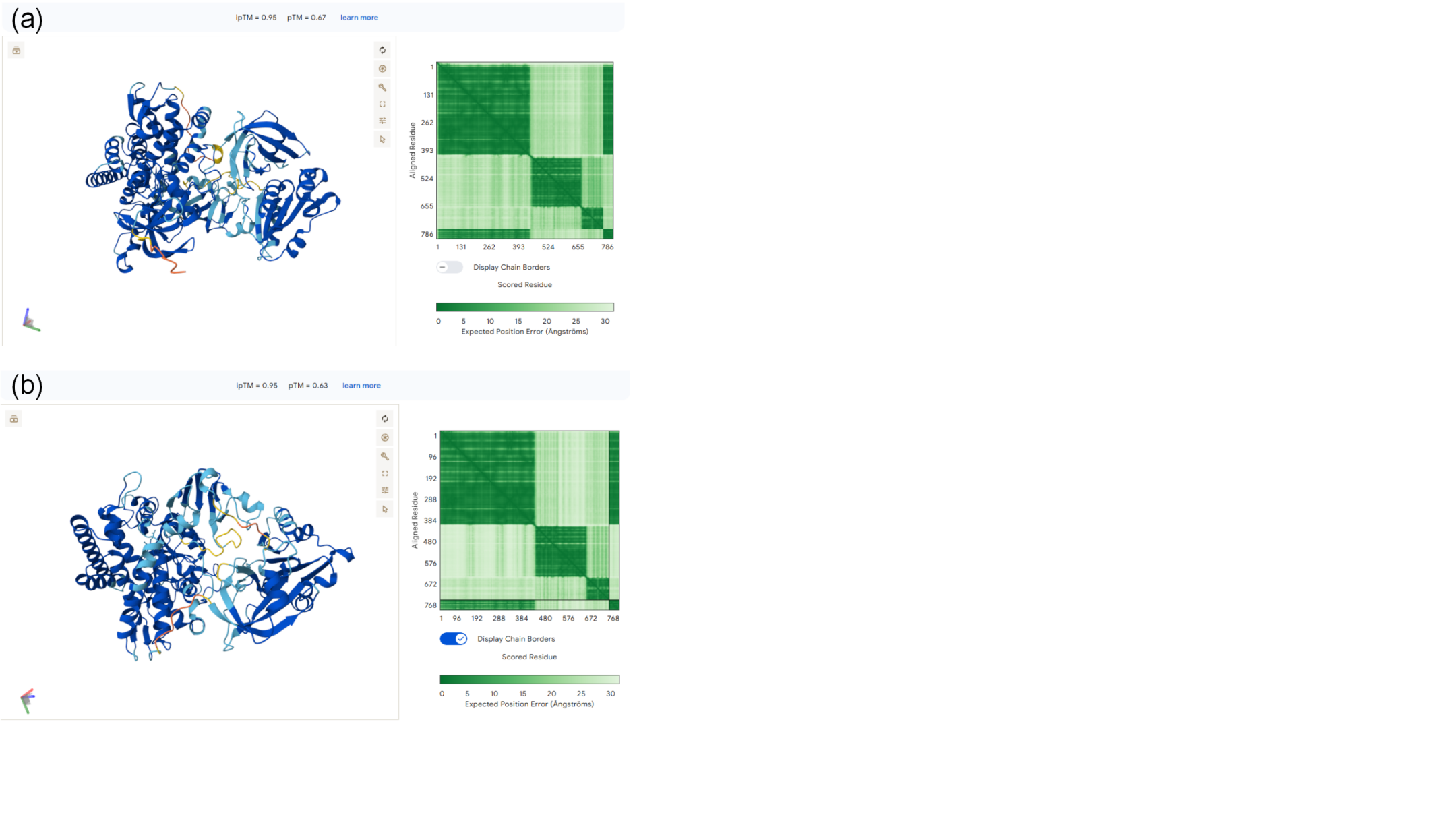


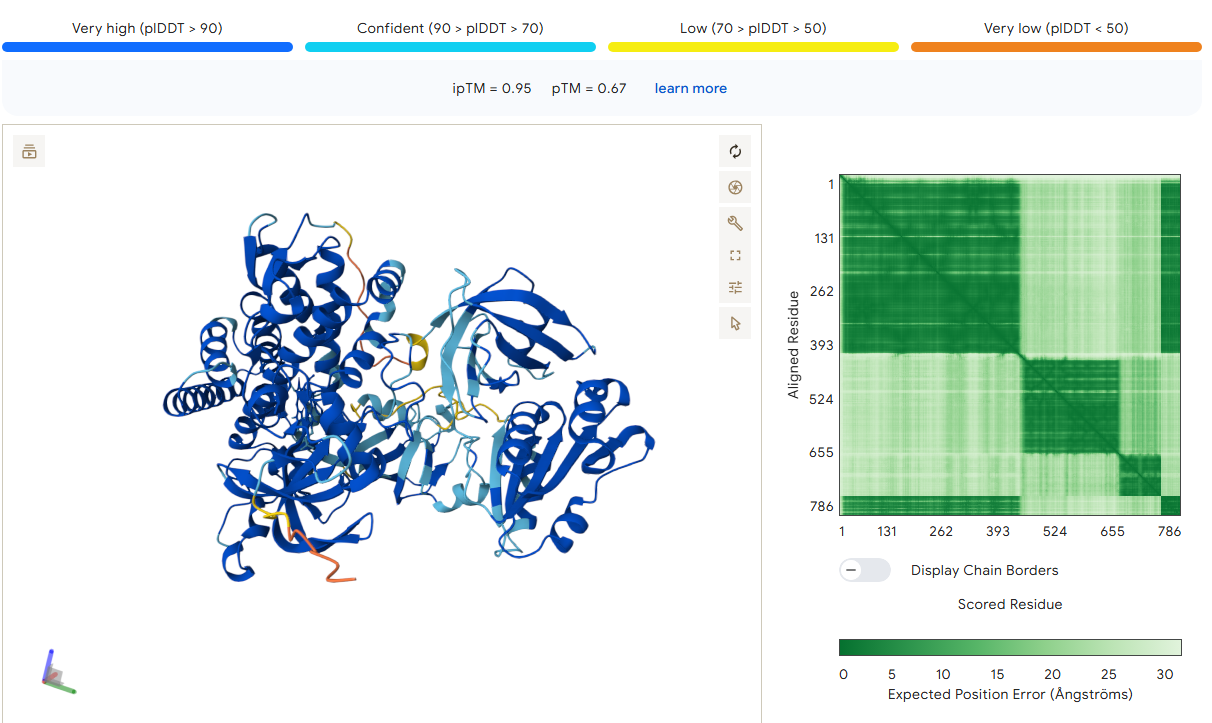


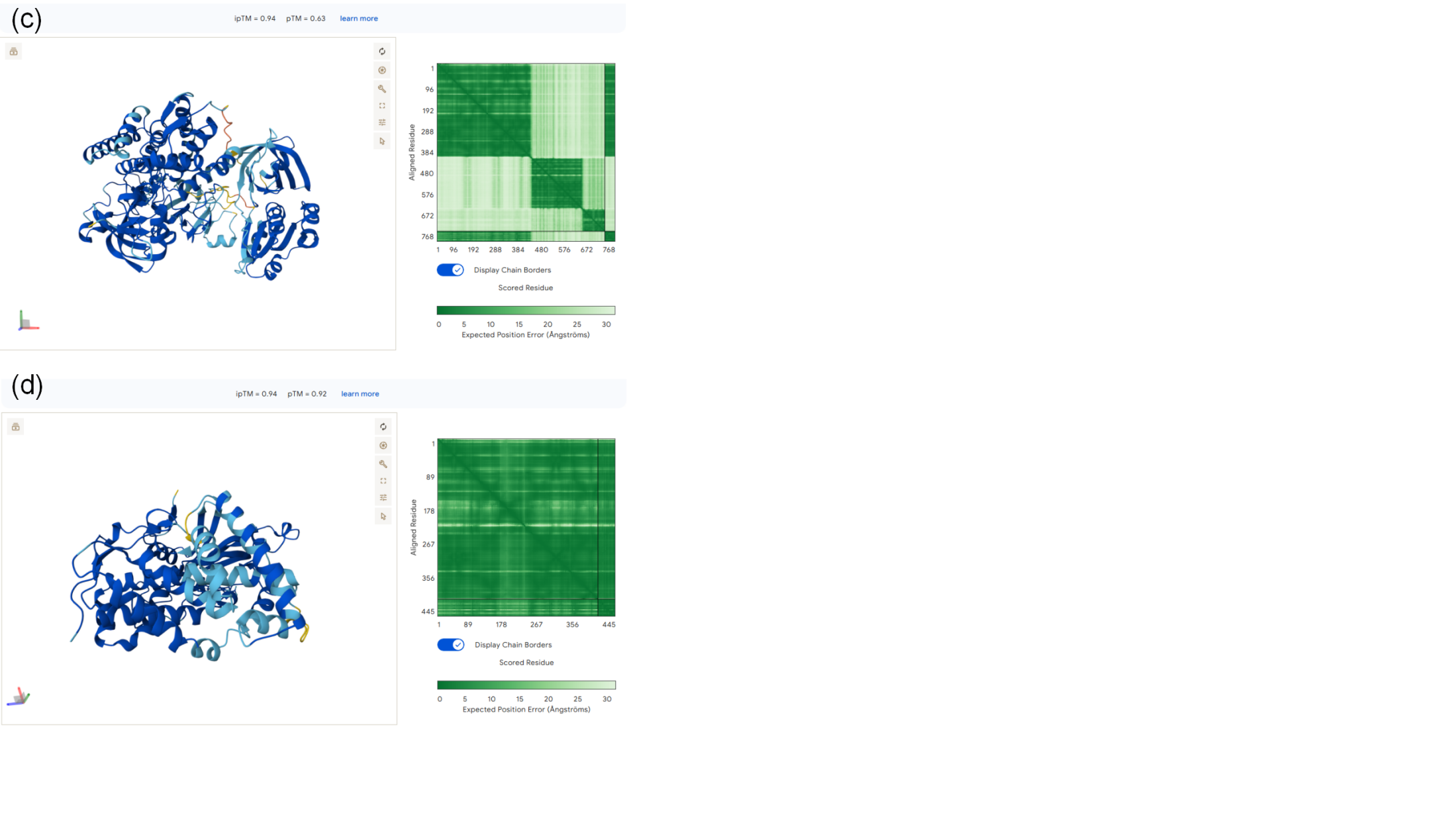


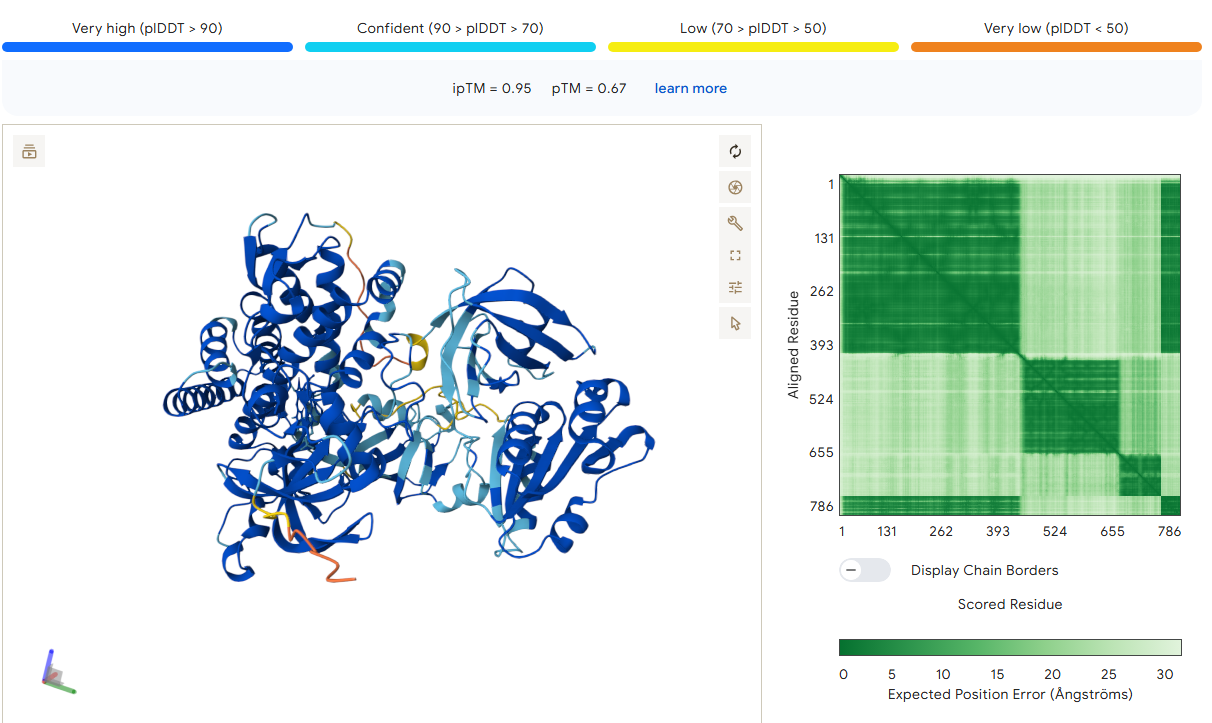


**Figure S4.** The predicted structures of full-length (a) VK1-CYP116B46-L13 (L13), (b) VK1-CYP116B46-L21 (L21), (c) VK1-CYP116B46-L21 F346K/R354M (L21-M2) and (d) the heme domain of VK1-CYP116B46-L21-P83A/A177M/K180F (L21-M3) were modeled and evaluated.


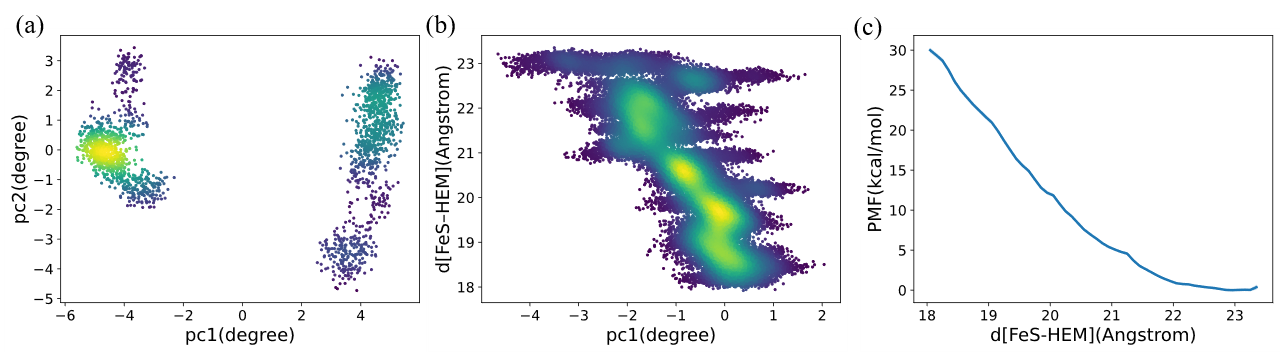


**Figure S5.** (a) PCA projection of the distal and proximal conformation MD simulations for L21-M2; (b) Projection of aggregated trajectories from umbrella sampling to PC1 and d[FeS–Heme]; (c) PMF as a function of d[FeS–Heme].


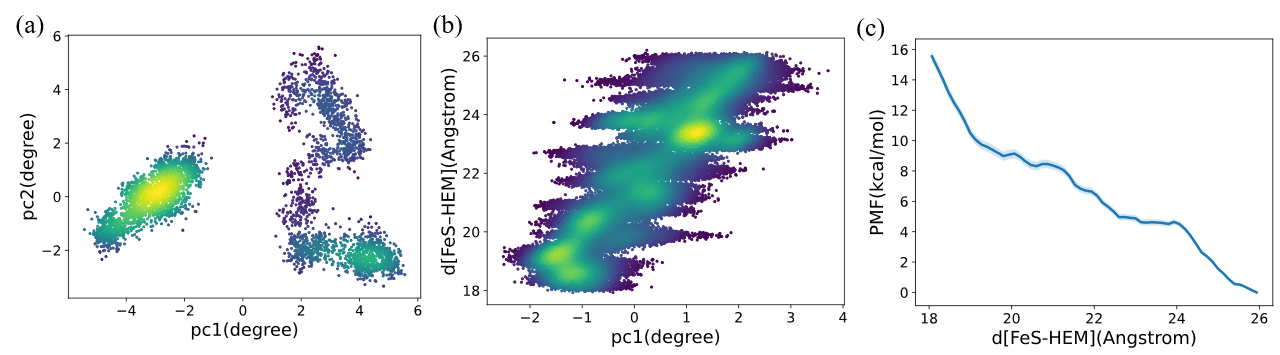


**Figure S6.** (a) PCA projection of the distal and proximal conformation MD simulations for L21; (b) Projection of aggregated trajectories from umbrella sampling to PC1 and d[FeS–Heme]; (c) PMF as a function of d[FeS–Heme].


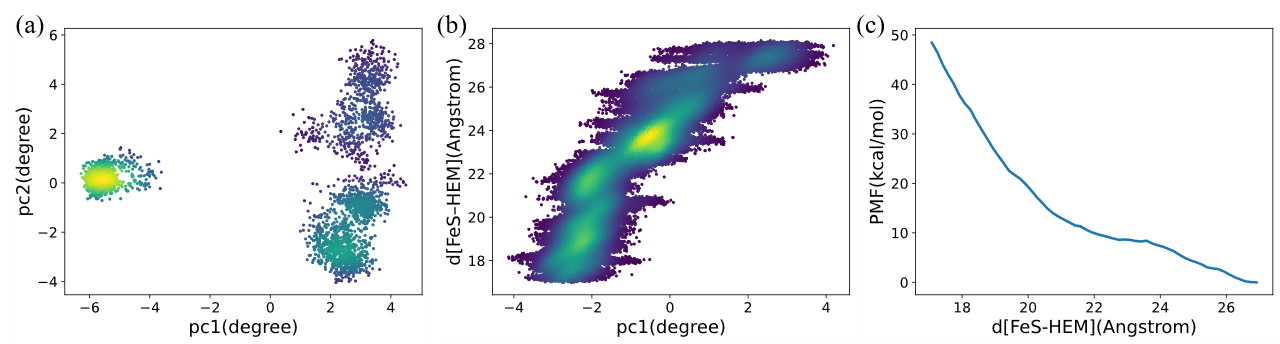


**Figure S7**. (a) PCA projection of the distal and proximal conformation MD simulations for L13; (b) Projection of aggregated trajectories from umbrella sampling to PC1 and d[FeS–Heme]; (c) PMF as a function of d[FeS–Heme].


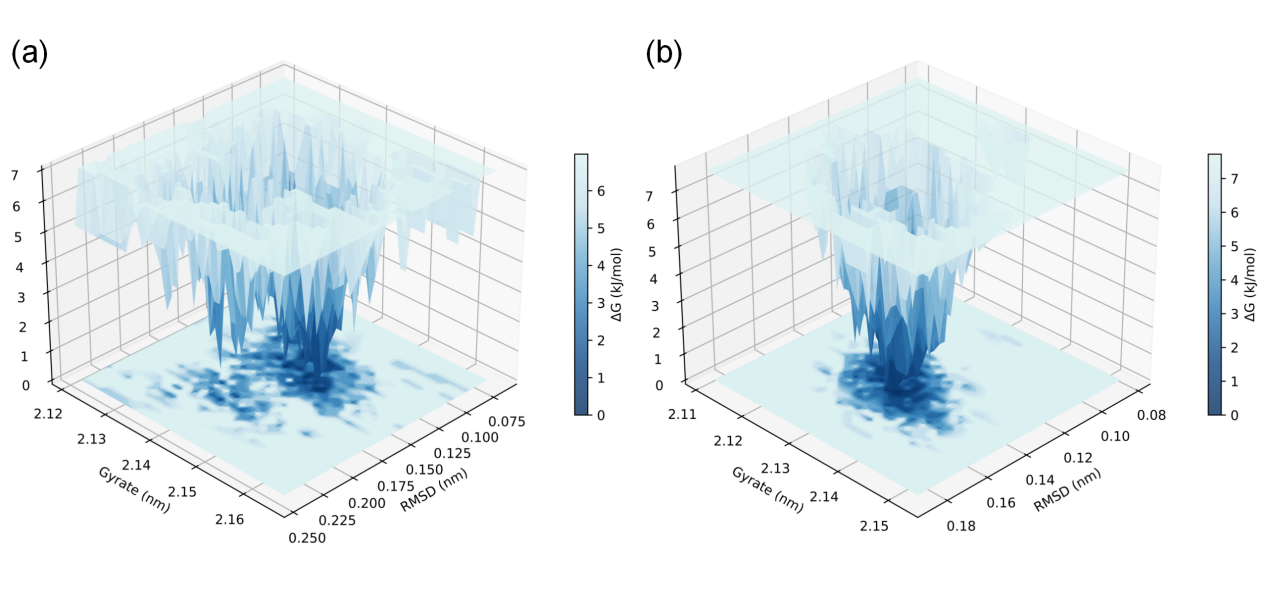


**Figure S8.** Free energy landscapes of (a) L21 and (b) L21-M3 derived from 200 ns MD simulations.


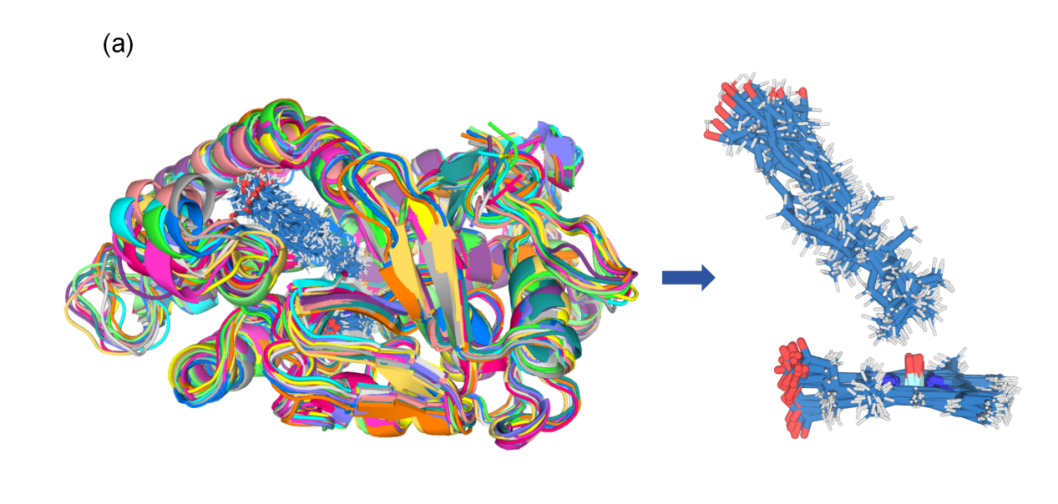


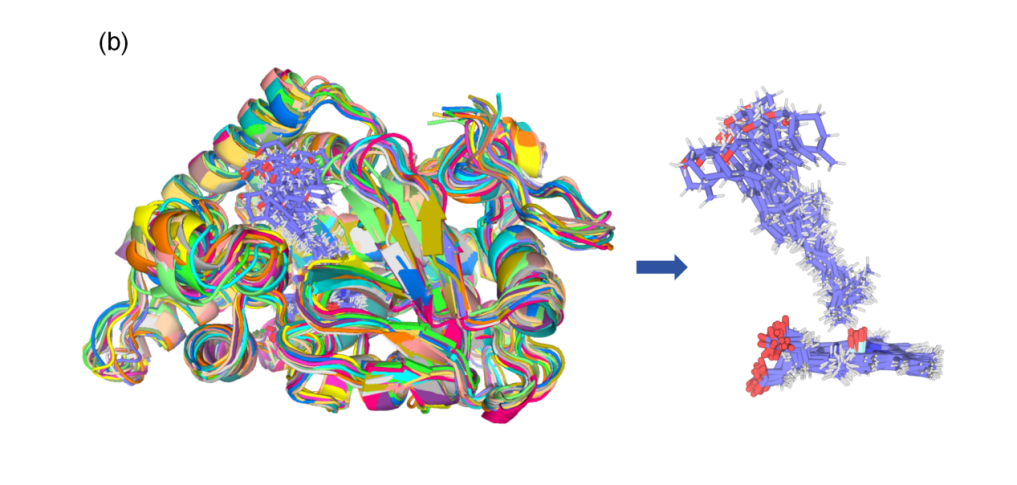


**Figure S9.** The lowest energy conformational clusters of L21(a) and L21-M3(b) extracted from the free energy landscapes.


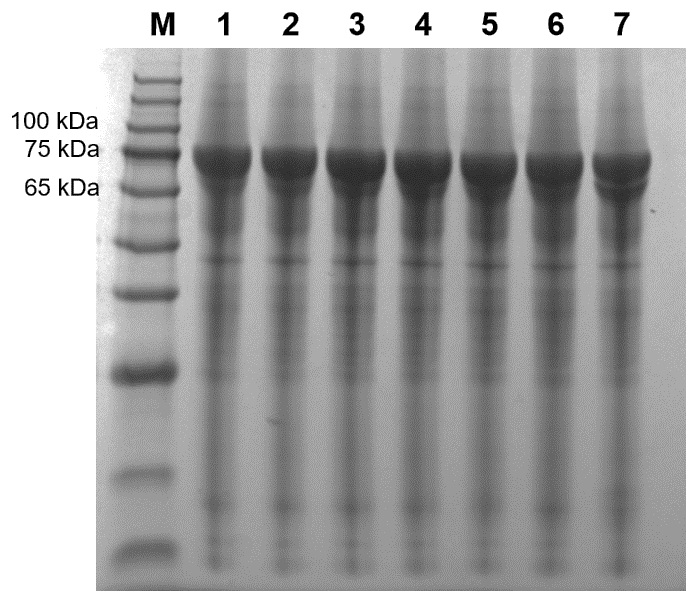


**Figure S10.** SDS-PAGE analysis of the expression of P450s. The proteins were purified by Ni-NTA column. M is the protein molecular weight, marker; Lane 1, VK1-CYP116B46-L13; Lanes 2, VK1-CYP116B46-L21; Lanes 3, VK1-CYP116B46-L21-R354M; Lanes 4, VK1-CYP116B46-L21-F346K/R354M (L21-M2); Lanes 5, VK1-CYP116B46-L21-K180F; Lanes 6, VK1-CYP116B46-L21-P83A/A177M/K180F (L21-M3); Lanes 7, VK1-CYP116B46-L21-P83A/A177M/K180F /F346K/R354M (L21-M5).


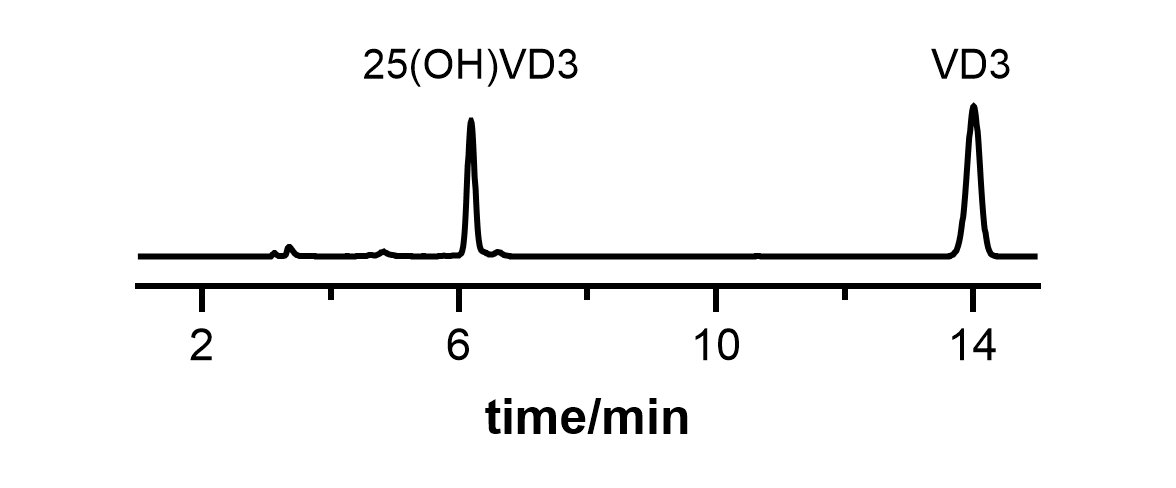


**Figure S11.** Typical HPLC chromatograms for mixed standards of VD3 and 25(OH)VD3.


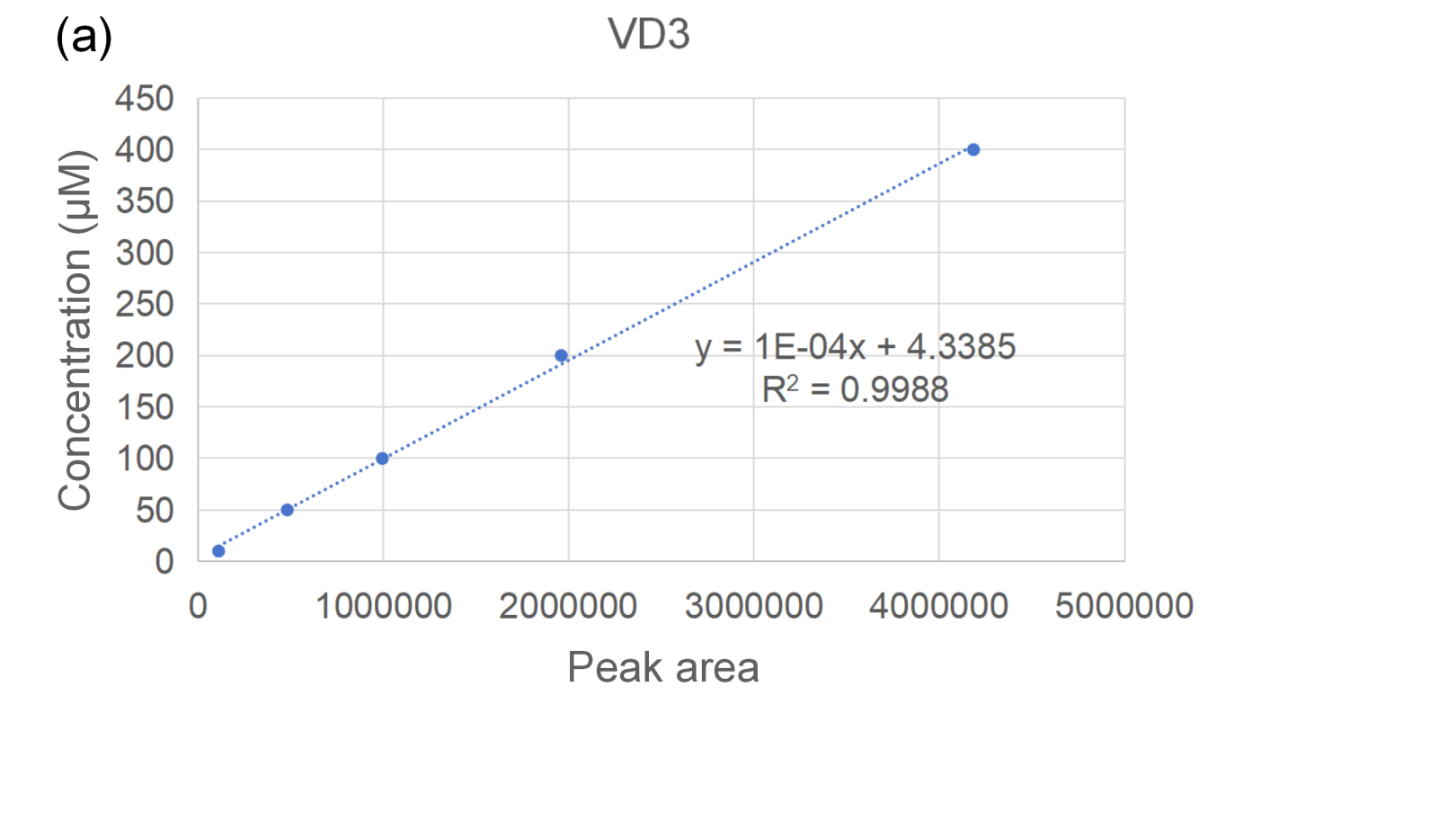


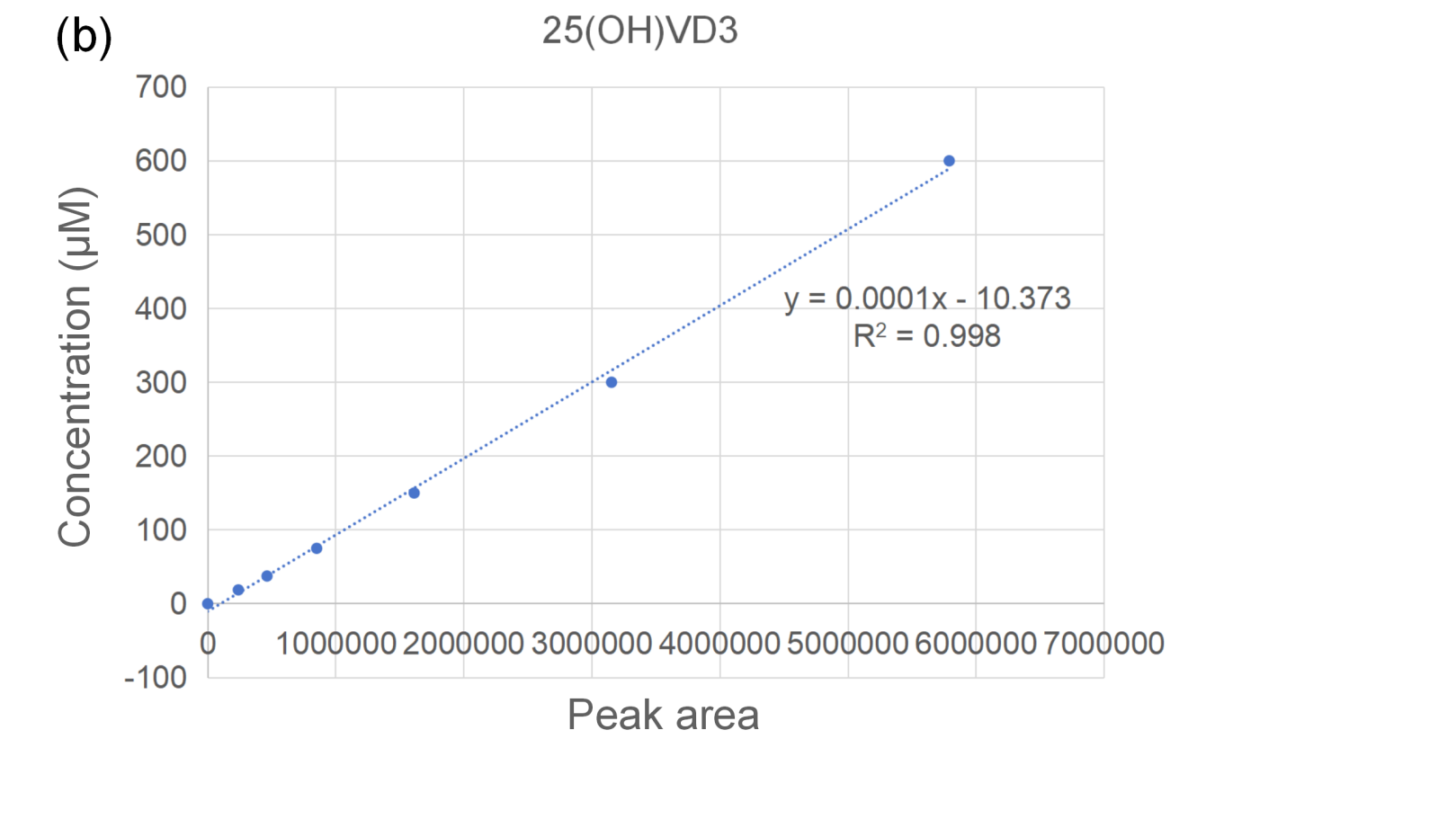


**Figure S12.** Regression curves of peak area versus concentration for (a) VD3 and (b) 25(OH)VD3 standards.


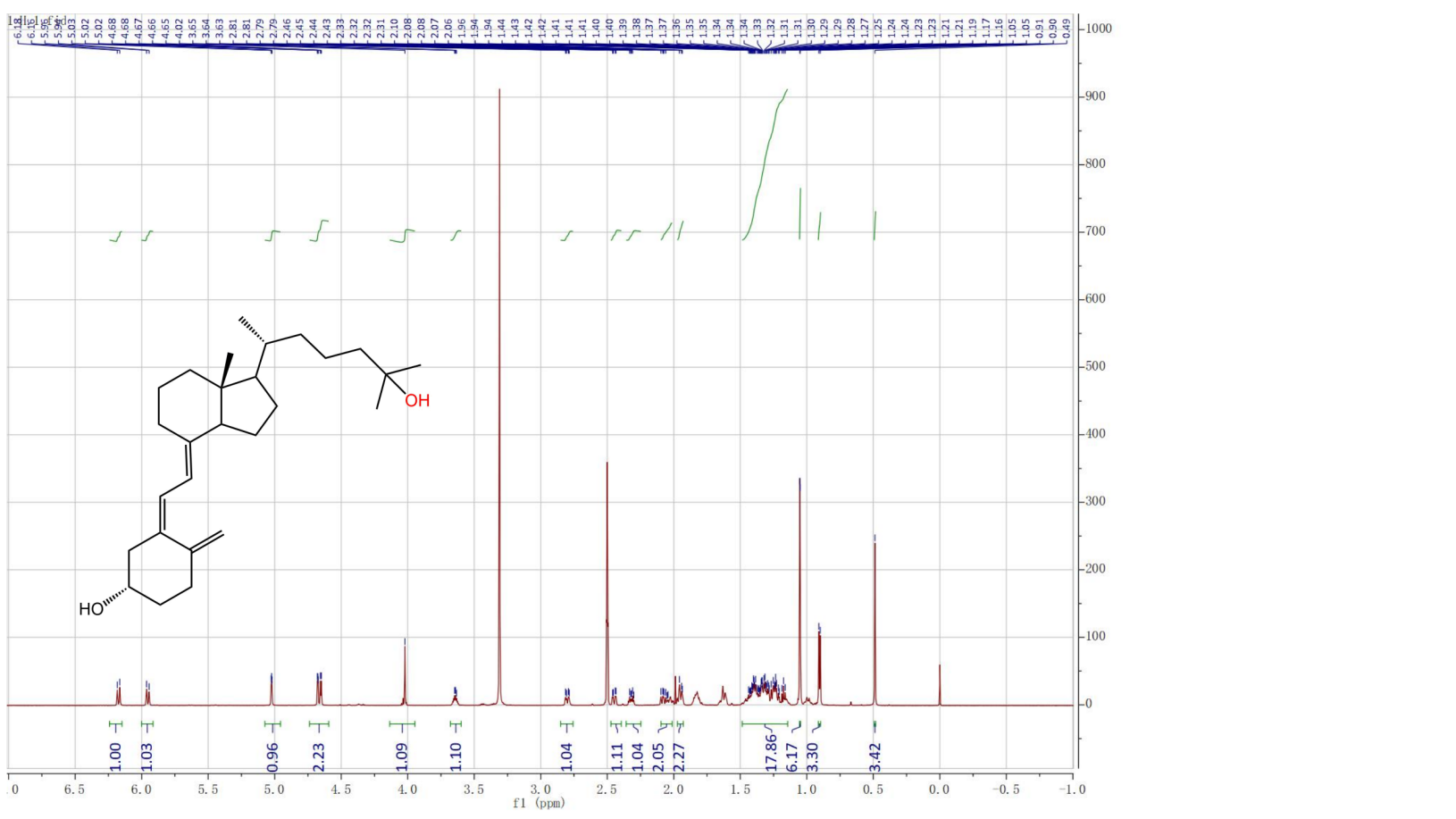


**Figure S13**. ^1^H-NMR spectrum of the prepared 25(OH)VD3 obtained from the biotransformation of VD3 in DMSO-*d*_6_ . ^1^H-NMR spectrum was recorded on Avance-600 (^1^H at 600 MHz) spectrometer. δ 6.17 (d, *J* = 11.2 Hz, 1H), 5.95 (d, *J* = 11.2 Hz, 1H), 5.06 – 4.94 (m, 1H), 4.71 – 4.59 (m, 2H), 4.02 (s, 1H), 3.65 – 3.59 (m, 1H), 2.80 (dd, *J* = 12.3, 4.3 Hz, 1H), 2.45 (dd, *J* = 11.9, 3.6 Hz, 1H), 2.34 – 2.24 (m, 1H), 2.10 – 2.03 (m, 1H), 1.95 (d, *J* = 9.9 Hz, 2H), 1.50 – 1.14 (m, 7H), 1.05 (d, *J* = 1.5 Hz, 6H), 0.90 (d, *J* = 6.4 Hz, 3H), 0.49 (s, 3H).


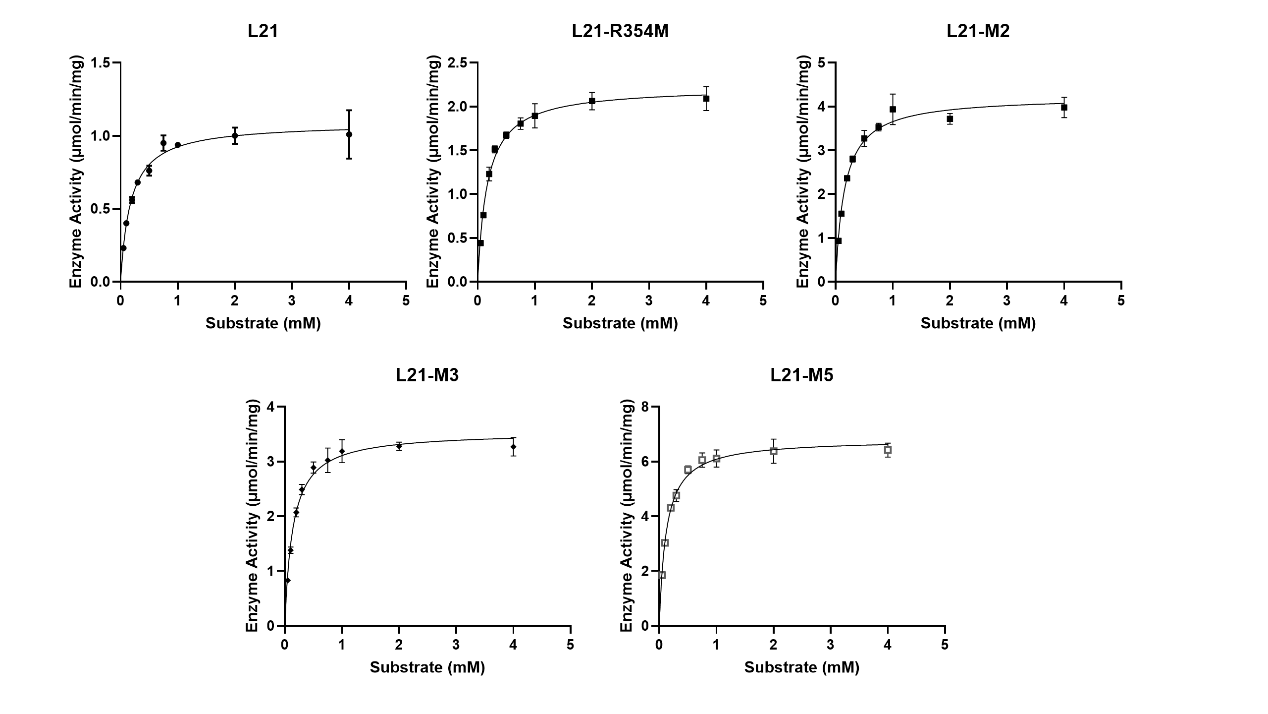


**Figure S14.** Kinetic parameters and fitting curves analysis of L21, L21-R354M, L21-M2, L21-M3 and L21-M5. Reaction conditions: purified P450s (1 μM) in PB (50 mM, pH 7.4), NADH 5 mM, VD3 0.01−5 mM, 25 °C, 1000 rpm for 10 min. The error bars indicate the standard deviation of three biological replicates (n = 3).
